# Supplementary material for: The Meta-Position of Phe4 in Leu-Enkephalin Regulates Potency, Selectivity, Functional Activity, and Signaling Bias at the Delta and Mu Opioid Receptors
Source: Molecules. 2019 Dec 12;24(24):4542. doi: 10.3390/molecules24244542 (PMC6943441; doi:10.3390/molecules24244542)
Supplement: Supplementary file 1 [file molecules-24-04542-s001.pdf]

Supporting Information for

The Meta-Position of Phe^4 in Leu-Enkephalin Regulates Potency, Selectivity, Functional Activity, and Bias at the Delta and Mu Opioid Receptors

Robert J. Cassell[1,†], Krishna K. Sharma[4,†], Hongyu Su[1], Benjamin R. Cummins[5], Haoyue Cui[6], Kendall L. Mores[1], Arryn T. Blaine[1], Ryan A. Altman[4,‡], Richard M. van Rijn[1,2,3,‡]

[1]Department of Medicinal Chemistry and Molecular Pharmacology, College of Pharmacy, Purdue University

[2]Purdue Institute for Drug Discovery, Purdue University

[3]Purdue Institute for Integrative Neuroscience, Purdue University

[4]Department of Medicinal Chemistry, The University of Kansas

[5]Department of Chemistry, Purdue University

[6]College of Wuya, Shenyang Pharmaceutical University

rvanrijn@purdue.edu, raaltman@ku.edu

Table of Contents

Table S1……………………………………………………………………….……….. SI-2

Table S2………………………………………………………………………………..SI-3

Characterization of Peptides SI-4

NMR Spectra of Peptides...SI-9

HPLC Chromatograms of Peptides...SI-24

Table S1: Efficacy of cAMP inhibiton of Meta-substituted Phe^{4} Analogs of Leu-enkephalin at δOR and µOR.

**cAMP**	**δOR**	**µOR**
Compound	Efficacy (%+SD)	Efficacy (%+SD)
**1a** (F)	95 ± 6	98 ± 6
**1b** (Cl)	94 ± 5	99 ± 7
**1c** (Br)	96 ± 4	104 ± 11
**1d** (I)	97 ± 4	94 ± 11
**1e** (Me)	92 ± 8	85 ± 10
**1f** (OMe)	98 ± 10	87 ± 5
**1g** (CF_3)	92 ± 8	90 ± 4
**1h** (CN)	100 ± 3	92 ± 13
**1i** (NO_2)	99 ± 6	105 ± 12
**1j** (2-pyr)	100 ± 8	99 ± 5
**1k** (3-pyr)	97 ± 8	100 ± 7
**1l** (4-pyr)	99 ± 10	99 ± 6
DADLE	97 ± 4	98 ± 6
Leu-enkephalin	100	100 ± 9
DAMGO	98 ± 4	100

Table S2: LogR values for Meta-substituted Phe[4] Analogs of Leu-enkephalin at δOR and μOR in the cAMP and β-arrestin 2 (β-arr2) assays. 95% confidence intervals are presented between parentheses.

	**LogR (δOR)**		**LogR (μOR)**	
Compound	cAMP	β-arr2	cAMP	β-arr2
**1a** (F)	9.4 (9.1-9.6)	8.1 (7.9-8.3)	8.0 (7.7-8.3)	5.8 (5.6-6.1)
**1b** (Cl)	10.6 (10.3-10.9)	9.0 (8.8-9.3)	7.5 (7.1-7.8)	7.2 (6.9-7.4)
**1c** (Br)	10.5 (10.2-10.9)	9.4 (9.1-9.6)	8.1 (7.8-8.5)	7.2 (6.9-7.4)
**1d** (I)	10.6 (10.3-10.9)	9.0 (8.8-9.3)	8.0 (7.6-8.4)	6.6 (6.4-6.9)
**1e** (Me)	10 (9.7-10.3)	8.3 (8.1-8.5)	8.1 (7.7-8.4)	6.1 (5.8-6.4)
**1f** (OMe)	9.8 (9.5-10.1)	7.9 (7.7-8.1)	7.4 (7.0-7.7)	5.6 (5.3-5.8)
**1g** (CF_3)	9.7 (9.4-10)	8.3 (8.1-8.6)	8.1 (7.8-8.4)	6.6 (6.4-6.9)
**1h** (CN)	9.3 (9.0-9.5)	7.4 (7.2-7.7)	7.5 (7.2-7.7)	5.9 (5.6-6.1)
**1i** (NO_2)	9.0 (8.8-9.3)	7.2 (6.9-7.4)	7.2 (6.9-7.5)	5.3 (5.0-5.5)
**1j** (2-pyr)	8.2 (8.0-8.5)	6.6 (6.3-6.9)	7.4 (7.1-7.7)	5.3 (5.0-5.6)
**1k** (3-pyr)	7.3 (7.0-7.6)	5.4 (5.1-5.8)	6.6 (6.3-6.9)	3.2 (2.7-3.6)
**1l** (4-pyr)	7.5 (7.3-7.8)	6.2 (5.9-6.4)	6.6 (6.3-6.8)	3.9 (3.6-4.2)
DADLE	9.0 (8.8-9.3)	8.3 (8.0-8.6)	7.6 (7.3-7.9)	6.4 (6.3-6.5)
Leu-enkephalin	8.9 (8.7-9.2)	7.8 (7.7-8.0)	7.0 (6.8-7.3)	5.5 (5.2-5.7)
DAMGO	5.9 (5.6-6.2)	-	7.8 (7.6-8.0)	6.9 (6.8-7.1)

SI-3 Characterization of Peptides

H_2N-Tyr-Gly-Gly-(*meta*-F)Phe-Leu-OH (1a). Yield 52.6%, 60.0 mg colorless solid. ^{1}H NMR (500 MHz, MeOD-d_4) δ 7.29 (q, J = 7.8 Hz, 1H), 7.16 – 7.06 (m, 4H), 6.95 (t, J = 8.6 Hz, 1H), 6.81 (d, J = 8.4 Hz, 2H), 4.74 (dd, J = 9.8, 4.6 Hz, 1H), 4.38 (t, J = 7.3 Hz, 1H), 4.15 – 4.09 (m, 1H), 4.00 – 3.91 (m, 2H), 3.80 – 3.72 (m, 2H), 3.24 – 3.14 (m, 2H), 3.03 – 2.98 (m, 2H), 1.77 – 1.61 (m, 3H), 0.96 (d, J = 6.2 Hz, 3H), 0.92 (d, J = 6.2 Hz, 3H). ^{13}C NMR (126 MHz, MeOD-d_4) δ 173.9, 171.9, 170.1, 169.8, 169.7, 162.8 (d, J = 244.0 Hz), 156.9, 139.8 (d, J = 7.6 Hz), 130.1, 129.6 (d, J = 8.2 Hz), 124.9 (d, J = 2.8 Hz), 124.6, 115.7 (d, J = 21.6 Hz), 115.5, 113.0 (d, J = 21.3 Hz), 54.7, 54.0, 51.4, 42.6, 41.8, 40.3, 37.2 (d, J = 1.8 Hz), 36.3, 24.5, 22.0, 20.5. ^{19}F NMR (471 MHz, MeOD-d_4) δ –115.0. HRMS (ESI$^+$) mass calculated for $[M+H]^+$ ($C_{28}H_{36}FN_5O_7$) m/z 574.2677, found m/z 574.2669; purity ≥95%, rt 2.76 min (protocol A).

H_2N-Tyr-Gly-Gly-(*meta*-Cl)Phe-Leu-OH (1b). Yield 50.9%, 60.2 mg colorless solid. ^{1}H NMR (500 MHz, MeOD-d_4) δ 7.37 (s, 1H), 7.31 – 7.20 (m, 3H), 7.13 (d, J = 8.4 Hz, 2H), 6.81 (d, J = 8.5 Hz, 2H), 4.72 (dd, J = 9.8, 4.6 Hz, 1H), 4.39 (t, J = 7.4 Hz, 1H), 4.18 – 4.06 (m, 1H), 4.00 – 4.10 (m, 2H), 3.80 – 3.73 (m, 2H), 3.78 (d, J = 22.5 Hz, 1H), 3.75 (d, J = 23.1 Hz, 1H), 3.21 – 3.16 (m, 2H), 3.02 – 2.96 (m, 2H), 1.76 – 1.59 (m, 3H), 0.97 (d, J = 6.2 Hz, 3H), 0.92 (d, J = 6.1 Hz, 3H). ^{13}C NMR (126 MHz, MeOD-d_4) δ 174.8, 171.9, 170.0, 169.8, 169.7, 156.9, 139.3, 133.7, 130.1, 129.5, 129.0, 127.5, 126.5, 124.5, 115.5, 54.7, 54.0, 51.3, 42.6, 41.8, 40.3, 37.1, 36.3, 24.5, 22.0, 20.4. HRMS (ESI$^+$) mass calculated for $[M+H]^+$ ($C_{28}H_{36}ClN_5O_7$) m/z 590.2473, found m/z 590.2431; purity ≥95%, rt 1.65 min (protocol B).

H_2N-Tyr-Gly-Gly-(*meta*-Br)Phe-Leu-OH (1c). Yield 47.6%, 60.5 mg colorless solid. ^{1}H NMR (500 MHz, MeOD-d_4) δ 7.54 (s, 1H), 7.38 (d, J = 7.9 Hz, 1H), 7.29 (d, J = 7.6 Hz, 1H), 7.21 (t, J = 7.8 Hz, 1H), 7.14 (d, J = 8.2 Hz, 2H), 6.81 (d, J = 8.2 Hz, 2H), 4.71 (dd, J = 10.1, 4.4 Hz, 1H), 4.33 (t, J = 7.3 Hz, 1H), 4.12 (t, J = 7.3 Hz, 1H), 3.99 (d, J = 26.6 Hz, 1H), 3.95 (d, J = 27.1 Hz, 1H), 3.77 (d, J = 24.5 Hz, 1H), 3.73 (d, J = 25.0 Hz, 1H), 3.19 (ddd, J = 15.3, 10.5, 5.4 Hz, 2H), 3.24 – 2.94 (m, 2H), 1.83 – 1.55 (m, 3H), 0.96 (d, J = 6.2 Hz, 3H), 0.92 (d, J = 6.1 Hz, 3H). ^{13}C NMR (126 MHz, MeOD-d_4) δ 171.8, 170.0, 170.0, 169.8, 156.9, 139.7, 132.0, 130.1, 129.7, 129.4, 127.9, 124.6, 121.9, 115.5, 54.8, 54.1, 52.0, 48.1, 47.9, 42.7, 41.8, 40.5, 37.0, 36.4, 24.6,

22.0, 20.5. HRMS (ESI$^+$) mass calculated for $[M+H]^+$ ($C_{28}H_{36}BrN_5O_7$) m/z 656.1696, found m/z 656.1711; purity ≥95%, rt 2.8 min (protocol A).

H_2N-Tyr-Gly-Gly-(*meta*-I)Phe-Leu-OH (1d). Yield 55.2%, 75.0 mg colorless solid. ^{1}H NMR (500 MHz, MeOD-d_4) δ 7.75 (s, 1H), 7.58 (d, *J* = 7.6 Hz, 1H), 7.33 (d, *J* = 7.7 Hz, 1H), 7.14 (d, *J* = 8.1 Hz, 2H), 7.07 (t, *J* = 7.8 Hz, 1H), 6.81 (d, *J* = 8.1 Hz, 2H), 4.68 (dd, *J* = 10.5, 4.1 Hz, 1H), 4.25 (dd, *J* = 9.1, 5.3 Hz, 1H), 4.12 (t, *J* = 7.2 Hz, 1H), 4.03 – 3.94 (m, 2H), 3.77 – 3.67 (m, 2H), 3.74 (d, *J* = 31.4 Hz, 1H), 3.70 (d, *J* = 32.0 Hz, 1H), 3.23 – 3.13 (m, 2H), 2.97 (ddd, *J* = 21.8, 14.0, 9.4 Hz, 2H), 1.76 – 1.54 (m, 3H), 0.96 (d, *J* = 6.1 Hz, 3H), 0.92 (d, *J* = 6.0 Hz, 3H). ^{13}C NMR (126 MHz, MeOD-d_4) δ 174.3, 171.7, 170.5, 170.1, 169.8, 156.9, 139.9, 138.0, 135.5, 130.1, 129.8, 128.4, 124.7, 115.5, 93.5, 54.8, 54.3, 53.2, 42.9, 41.8, 40.9, 36.9, 36.5, 24.7, 22.2, 20.7. HRMS (ESI$^+$) mass calculated for $[M+H]^+$ ($C_{28}H_{36}IN_5O_7$) m/z 682.1737, found m/z 681.1741; purity ≥95%, rt 2.97 min (protocol A).

H_2N-Tyr-Gly-Gly-(*meta*-Me)Phe-Leu-OH (1e). Yield 78.9%, 90.2 mg colorless solid. ^{1}H NMR (500 MHz, MeOD-d_4) δ 7.20 – 7.11 (m, 4H), 7.08 (d, *J* = 7.7 Hz, 1H), 7.03 (d, *J* = 7.5 Hz, 1H), 6.81 (d, *J* = 8.2 Hz, 2H), 4.69 (dd, *J* = 9.8, 4.6 Hz, 1H), 4.37 (t, *J* = 7.3 Hz, 1H), 4.12 (t, *J* = 7.3 Hz, 1H), 4.02 – 3.88 (m, 2H), 3.77 (d, *J* = 27.8 Hz, 1H), 3.74 (d, *J* = 28.2 Hz, 1H), 3.17 (ddd, *J* = 14.4, 9.3, 5.5 Hz, 2H), 2.97 (ddd, *J* = 23.4, 14.0, 9.0 Hz, 2H), 1.73 – 1.63 (m, 3H), 0.96 (d, *J* = 6.3 Hz, 3H), 0.92 (d, *J* = 6.2 Hz, 3H). ^{13}C NMR (126 MHz, MeOD-d_4) δ 175.2, 172.2, 170.0, 169.8, 169.7, 156.9, 137.7, 136.8, 130.1, 129.6, 127.9, 127.0, 126.0, 124.6, 115.4, 54.8, 54.5, 51.6, 42.6, 41.8, 40.4, 37.4, 36.3, 24.5, 22.0, 20.5, 20.0. HRMS (ESI$^+$) mass calculated for $[M+H]^+$ ($C_{29}H_{39}N_5O_7$) m/z 570.2927, found m/z 570.2923; purity ≥95%, rt 2.88 min (protocol A).

H_2N-Tyr-Gly-Gly-(*meta*-OMe)Phe-Leu-OH (1f). Yield 22.3%, 26.0 mg colorless solid. ^{1}H NMR (500 MHz, MeOD-d_4) δ 7.19 (t, *J* = 7.9 Hz, 1H), 7.13 (d, *J* = 8.3 Hz, 2H), 6.91 – 6.85 (m, 2H), 6.84 – 6.74 (m, 3H), 4.73 (dd, *J* = 9.6, 4.7 Hz, 1H), 4.43 (t, *J* = 7.4 Hz, 1H), 4.13 – 4.08 (m, 1H), 4.00 – 3.92 (m, 2H), 3.83 – 3.71 (m, 5H), 3.20-3.15 (m, 2H), 3.06 – 2.91 (m, 2H), 1.75 – 1.62 (m, 3H), 0.97 (d, *J* = 6.3 Hz, 3H), 0.92 (d, *J* = 6.4 Hz, 3H). ^{13}C NMR (126 MHz, MeOD-d_4) δ 174.2, 172.3, 170.0, 169.7, 169.6, 159.8, 156.9, 138.4, 130.1, 128.9, 124.5, 121.3, 115.4, 114.3, 112.1, 54.7, 54.2, 54.2, 50.7, 42.5, 41.8, 40.1, 37.5, 36.2, 24.5, 21.9, 20.4. HRMS (ESI$^+$) mass

calculated for $[M+H]^+$ ($C_{29}H_{39}N_5O_8$) m/z 586.2877, found m/z 585.2932; purity ≥95%, rt 1.57 min (protocol B).

H_2N-Tyr-Gly-Gly-(*meta*-CF_3)Phe-Leu-OH (1g). Yield 32.1%, 40.1 mg colorless solid. ^1H NMR (500 MHz, MeOD-d_4) δ 7.67 (s, 1H), 7.60 – 7.46 (m, 3H), 7.13 (d, J = 8.2 Hz, 2H), 6.80 (d, J = 8.2 Hz, 2H), 4.76 (dd, J = 9.8, 4.7 Hz, 1H), 4.41 (t, J = 7.4 Hz, 1H), 4.14 – 4.11 (m, 1H), 3.95 (t, J = 16.2 Hz, 1H), 3.76 (dd, J = 32.2, 16.8 Hz, 1H), 3.29 (dd, J = 14.0, 4.7 Hz, 1H), 3.18 (dd, J = 14.2, 6.5 Hz, 1H), 3.08 (dd, J = 14.0, 9.8 Hz, 1H), 3.00 (dd, J = 14.2, 8.1 Hz, 1H), 1.77 – 1.62 (m, 3H), 0.97 (d, J = 6.5 Hz, 1H), 0.92 (d, J = 6.2 Hz, 1H). ^{13}C NMR (126 MHz, MeOD-d_4) δ 174.5, 171.9, 170.0, 169.8, 169.7, 156.9, 138.4, 132.9, 130.2 (d, J = 31.8 Hz), 130.1, 128.7, 125.7 (q, J = 3.7 Hz), 124.5, 124.3 (q, J = 272.2 Hz), 123.1 (q, J = 4.0 Hz), 115.4, 54.7, 54.0, 51.0, 42.6, 41.7, 40.2, 37.3, 36.3, 24.5, 21.9, 20.4. ^{19}F NMR (471 MHz, MeOD-d_4) δ –63.3. HRMS (ESI^+) mass calculated for $[M+H]^+$ ($C_{29}H_{36}F_3N_5O_7$) m/z 624.2645, found m/z 624.2618; purity ≥95%, rt 2.89 min (protocol A).

H_2N-Tyr-Gly-Gly-(*meta*-CN)Phe-Leu-OH (1h). Yield 43.1%, 50.0 mg colorless solid. ^1H NMR (500 MHz, MeOD-d_4) δ 7.70 (s, 1H), 7.63 – 7.59 (m, 2H), 7.48 (t, J = 7.7 Hz, 1H), 7.14 (d, J = 8.0 Hz, 2H), 6.81 (d, J = 8.0 Hz, 2H), 4.75 (dd, J = 9.6, 4.8 Hz, 1H), 4.38 (dd, J = 9.0, 5.4 Hz, 1H), 4.13 (t, J = 7.4 Hz, 1H), 4.00-3.91 (m, 2H), 3.81-3.71 (m, 2H), 3.26 (dd, J = 14.0, 4.8 Hz, 1H), 3.19 (dd, J = 14.2, 6.4 Hz, 1H), 3.08-2.99 (m, 2H), 1.78 – 1.58 (m, 3H), 0.97 (d, J = 6.1 Hz, 3H), 0.92 (d, J = 6.0 Hz, 3H). ^{13}C NMR (126 MHz, MeOD-d_4) δ 173.9, 171.6, 170.0, 169.9, 169.7, 156.9, 138.8, 134.0, 132.8, 130.2, 130.1, 129.1, 124.6, 118.5, 115.5, 111.9, 54.8, 53.8, 51.5, 42.7, 41.8, 40.3, 37.0, 36.3, 24.6, 22.0, 20.4. HRMS (ESI^+) mass calculated for $[M+H]^+$ ($C_{29}H_{36}N_6O_7$) m/z 581.2723, found m/z 581.2755; purity ≥95%, rt 1.56 min (protocol B).

H_2N-Tyr-Gly-Gly-(*meta*-NO_2)Phe-Leu-OH (1i). Yield 66.7%, 80.3 mg colorless solid. ^1H NMR (400 MHz, MeOD-d_4) δ 8.30 (s, 1H), 8.11 (d, J = 8.2 Hz, 1H), 7.73 (d, J = 7.7 Hz, 1H), 7.54 (t, J = 7.9 Hz, 1H), 7.15 (d, J = 8.5 Hz, 2H), 6.81 (d, J = 8.5 Hz, 2H), 4.77 (dd, J = 10.6, 4.2 Hz, 1H), 4.25 (dd, J = 8.9, 5.5 Hz, 1H), 4.15 (dd, J = 8.3, 6.2 Hz, 1H), 3.96 (dd, J = 29.6, 16.7 Hz, 2H), 3.69 (dd, J = 45.1, 16.8 Hz, 2H), 3.37 (dd, J = 13.9, 4.3 Hz, 1H), 3.24 – 3.10 (m, 2H), 3.01 (dd, J = 14.2, 8.3 Hz, 1H), 1.73 – 160 (m, 3H), 0.96 (d, J = 6.1 Hz, 3H), 0.92 (d, J = 6.0 Hz, 3H). ^{13}C NMR (126 MHz, MeOD-d_4) δ 177.2, 171.4, 170.6, 170.0, 169.8, 156.9, 148.2, 139.5, 135.5, 130.1, 129.1, 124.7, 124.1, 121.3, 115.5, 54.9, 54.0, 53.2, 43.0, 41.8, 40.9, 37.0, 36.5,

24.7, 22.1, 20.6. HRMS (ESI$^+$) mass calculated for $[M+H]^+$ ($C_{29}H_{36}N_6O_9$) m/z 601.2606, found m/z 601.2622; purity ≥95%, rt 1.59 min (protocol B).

H_2N-Tyr-Gly-Gly-(2-pyridyl)Ala-Leu-OH (1j). Yield 10.8%, 12.1 mg colorless solid. ^1H NMR (500 MHz, MeOD-d_4) δ 8.63 (d, J = 5.4 Hz, 1H), 8.18 (t, J = 7.8 Hz, 1H), 7.73 (d, J = 7.9 Hz, 1H), 7.68 – 7.61 (m, 1H), 7.13 (d, J = 8.3 Hz, 2H), 6.80 (d, J = 8.2 Hz, 2H), 4.93 (dd, J = 8.5, 5.5 Hz, 1H), 4.44 – 4.38 (m, 1H), 4.11 (t, J = 7.2 Hz, 1H), 4.00 – 3.76 (m, 4H), 3.50 (dd, J = 14.3, 5.4 Hz, 1H), 3.38 – 3.27 (m, 1H), 3.17 (dd, J = 14.2, 6.5 Hz, 1H), 3.01 (dd, J = 14.2, 8.0 Hz, 1H), 1.78 – 1.58 (m, 3H), 0.97 (d, J = 6.0 Hz, 3H), 0.92 (d, J = 6.0 Hz, 3H). ^{13}C NMR (126 MHz, MeOD-d_4) δ 174.1, 170.8, 170.2, 169.8, 169.6, 156.9, 154.5, 144.7, 142.1, 130.1, 126.5, 124.5, 123.8, 115.4, 54.7, 52.4, 50.9, 42.4, 41.9, 39.9, 37.4, 36.2, 24.5, 21.9, 20.3. HRMS (ESI$^+$) mass calculated for $[M+H]^+$ ($C_{27}H_{36}N_6O_7$) m/z 557.2723, found m/z 557.2748; purity ≥95%, rt 2.46 min (protocol A).

H_2N-Tyr-Gly-Gly-(3-pyridyl)Ala-Leu-OH (1k). Yield 62.9%, 70.0 mg colorless solid. ^1H NMR (400 MHz, MeOD-d_4) δ 8.49 (s, 1H), 8.42 (d, J = 3.8 Hz, 1H), 7.83 (d, J = 7.9 Hz, 1H), 7.40 (dd, J = 7.8, 5.0 Hz, 1H), 7.14 (d, J = 8.5 Hz, 2H), 6.81 (d, J = 8.5 Hz, 2H), 4.77 (dd, J = 9.3, 4.9 Hz, 1H), 4.45 – 4.33 (m, 1H), 4.12 (dd, J = 8.2, 6.4 Hz, 1H), 3.95 (dd, J = 27.3, 16.7 Hz, 2H), 3.78 (dd, J = 19.3, 16.7 Hz, 2H), 3.28 – 3.16 (m, 2H), 3.09 – 2.97 (m, 2H), 1.77 – 1.57 (m, 3H), 0.97 (d, J = 6.2 Hz, 3H), 0.92 (d, J = 6.1 Hz, 3H). ^{13}C NMR (126 MHz, MeOD-d_4) δ 174.6, 173.8, 171.5, 170.1, 169.7, 156.9, 149.2, 146.7, 138.3, 133.6, 130.1, 124.5, 123.8, 115.5, 54.7, 53.5, 51.1, 42.5, 41.8, 40.2, 36.3, 34.7, 24.6, 21.9, 20.4. HRMS (ESI$^+$) mass calculated for $[M+H]^+$ ($C_{27}H_{36}N_6O_7$) m/z 557.2723, found m/z 557.2659; purity ≥95%, rt 2.46 min (protocol A).

H_2N-Tyr-Gly-Gly-(4-pyridyl)Ala-Leu-OH (1l). Yield 32.4%, 36.1 mg colorless solid. ^1H NMR (500 MHz, MeOD-d_4) δ 8.51 (d, J = 5.3 Hz, 2H), 7.53 (d, J = 5.3 Hz, 2H), 7.13 (d, J = 8.2 Hz, 2H), 6.80 (d, J = 8.3 Hz, 2H), 4.84 (dd, J = 9.4, 4.9 Hz, 1H), 4.45 – 4.36 (m, 1H), 4.13 (dd, J = 8.1, 6.4 Hz, 1H), 3.94 (dd, J = 20.4, 16.7 Hz, 2H), 3.78 (dd, J = 30.3, 16.7 Hz, 2H), 3.33 – 3.27 (m, 1H), 3.21 – 3.08 (m, 2H), 3.01 (dd, J = 14.2, 8.1 Hz, 1H), 1.75 – 1.63 (m, 3H), 0.97 (d, J = 6.1 Hz, 3H), 0.92 (d, J = 6.1 Hz, 3H). ^{13}C NMR (126 MHz, MeOD-d_4) δ 174.3, 173.8, 171.2, 170.1, 169.7, 156.9, 150.2, 146.7, 130.1, 125.8, 124.5, 115.4, 54.7, 52.9, 50.9, 42.6, 41.8, 40.0,

37.1, 36.2, 24.5, 21.9, 20.3. HRMS (ESI$^+$) mass calculated for $[M+H]^+$ ($C_{27}H_{36}N_6O_7$) m/z 557.2723, found m/z 557.2729; purity ≥95%, rt 1.20 min (protocol B).

NMR Spectra of Peptides

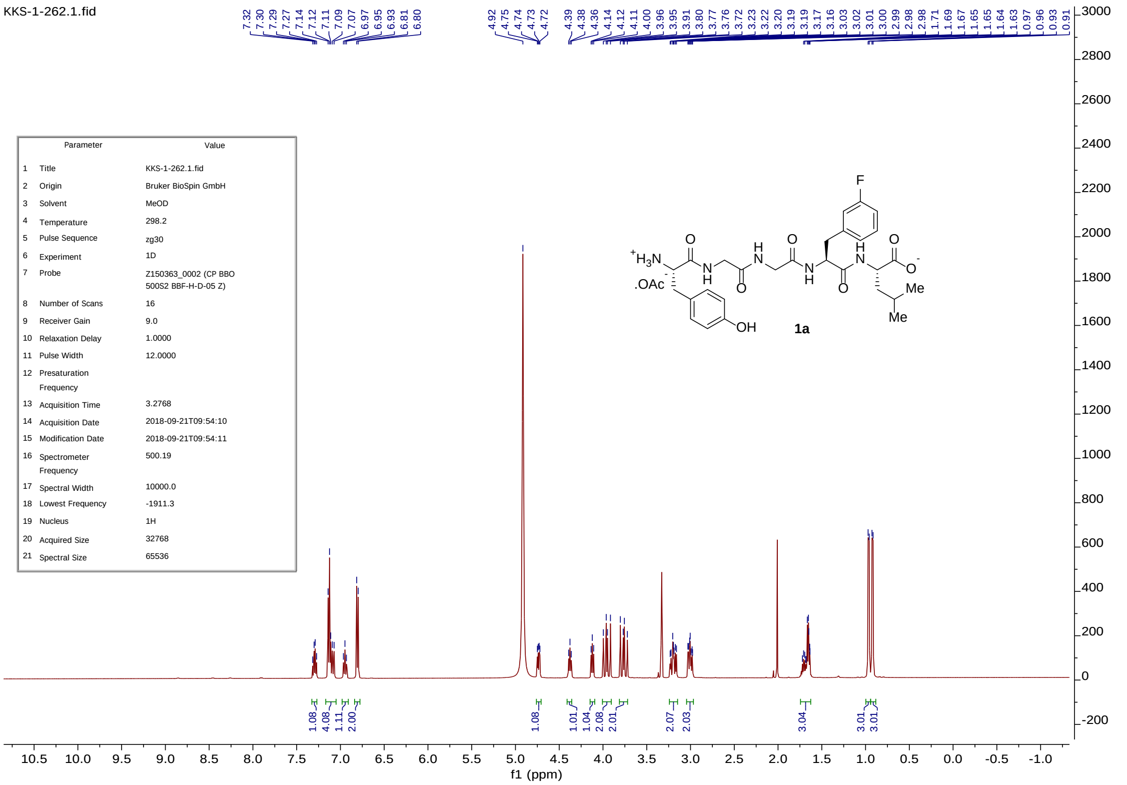

	Parameter	Value
1	Title	KKS-1-262.1.fid
2	Origin	Bruker BioSpin GmbH
3	Solvent	MeOD
4	Temperature	298.2
5	Pulse Sequence	zg30
6	Experiment	1D
7	Probe	Z150363_0002 (CP BBO 500S2 BBF-H-D-05 Z)
8	Number of Scans	16
9	Receiver Gain	9.0
10	Relaxation Delay	1.0000
11	Pulse Width	12.0000
12	Presaturation Frequency	
13	Acquisition Time	3.2768
14	Acquisition Date	2018-09-21T09:54:10
15	Modification Date	2018-09-21T09:54:11
16	Spectrometer Frequency	500.19
17	Spectral Width	10000.0
18	Lowest Frequency	-1911.3
19	Nucleus	1H
20	Acquired Size	32768
21	Spectral Size	65536

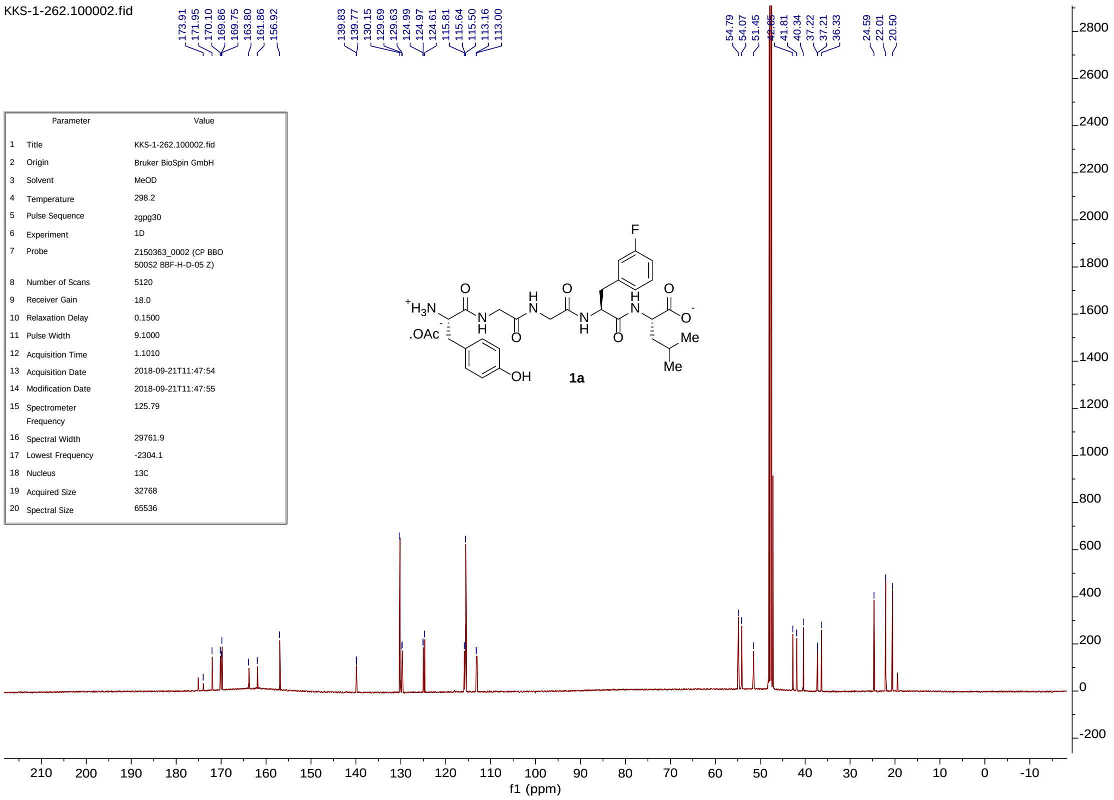

	Parameter	Value
1	Title	KKS-1-262.100002.fid
2	Origin	Bruker BioSpin GmbH
3	Solvent	MeOD
4	Temperature	298.2
5	Pulse Sequence	zgpg30
6	Experiment	1D
7	Probe	Z150363_0002 (CP BBO 500S2 BBF-H-D-05 Z)
8	Number of Scans	5120
9	Receiver Gain	18.0
10	Relaxation Delay	0.1500
11	Pulse Width	9.1000
12	Acquisition Time	1.1010
13	Acquisition Date	2018-09-21T11:47:54
14	Modification Date	2018-09-21T11:47:55
15	Spectrometer Frequency	125.79
16	Spectral Width	29761.9
17	Lowest Frequency	-2304.1
18	Nucleus	13C
19	Acquired Size	32768
20	Spectral Size	65536

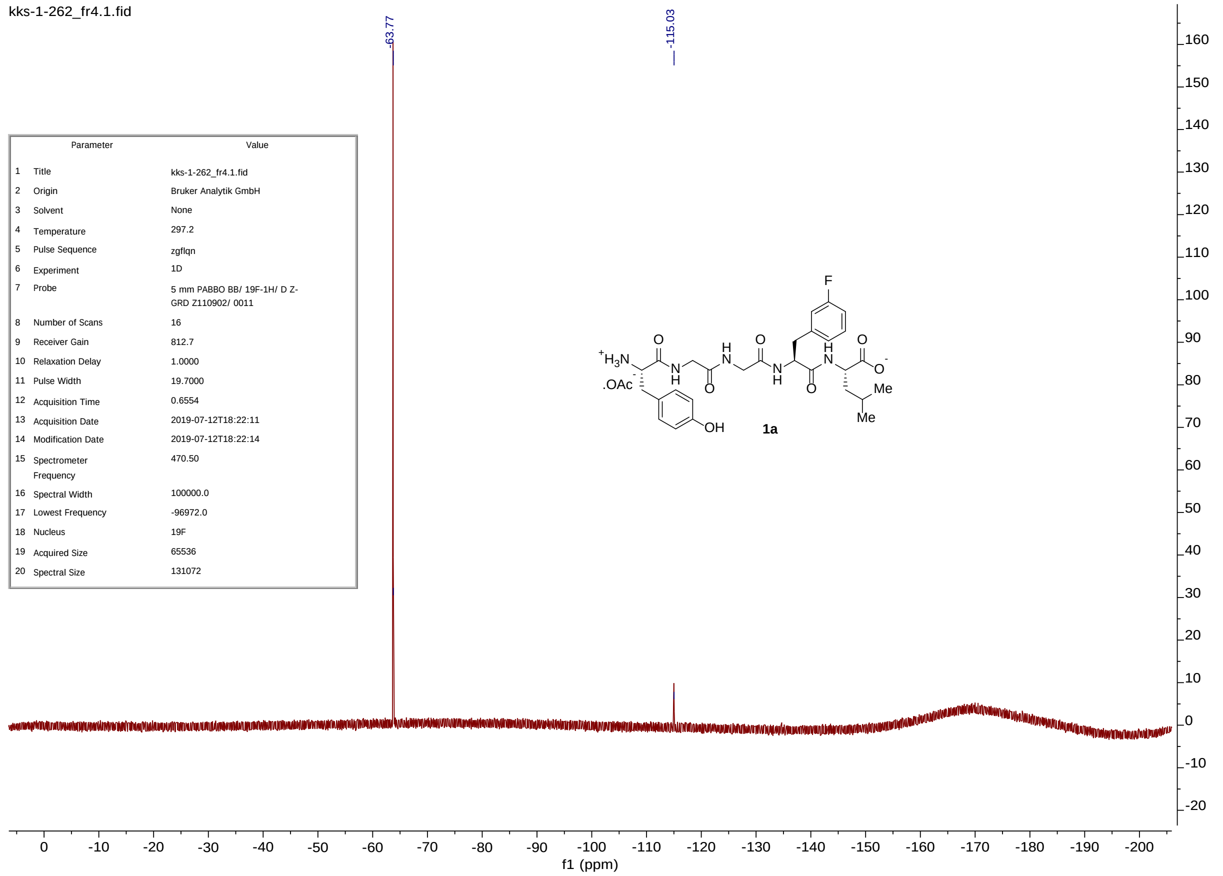

	Parameter	Value
1	Title	kks-1-262_fr4.1.fid
2	Origin	Bruker Analytik GmbH
3	Solvent	None
4	Temperature	297.2
5	Pulse Sequence	zgflqn
6	Experiment	1D
7	Probe	5 mm PABBO BB/ 19F-1H/ D Z-GRD Z110902/ 0011
8	Number of Scans	16
9	Receiver Gain	812.7
10	Relaxation Delay	1.0000
11	Pulse Width	19.7000
12	Acquisition Time	0.6554
13	Acquisition Date	2019-07-12T18:22:11
14	Modification Date	2019-07-12T18:22:14
15	Spectrometer Frequency	470.50
16	Spectral Width	100000.0
17	Lowest Frequency	-96972.0
18	Nucleus	19F
19	Acquired Size	65536
20	Spectral Size	131072

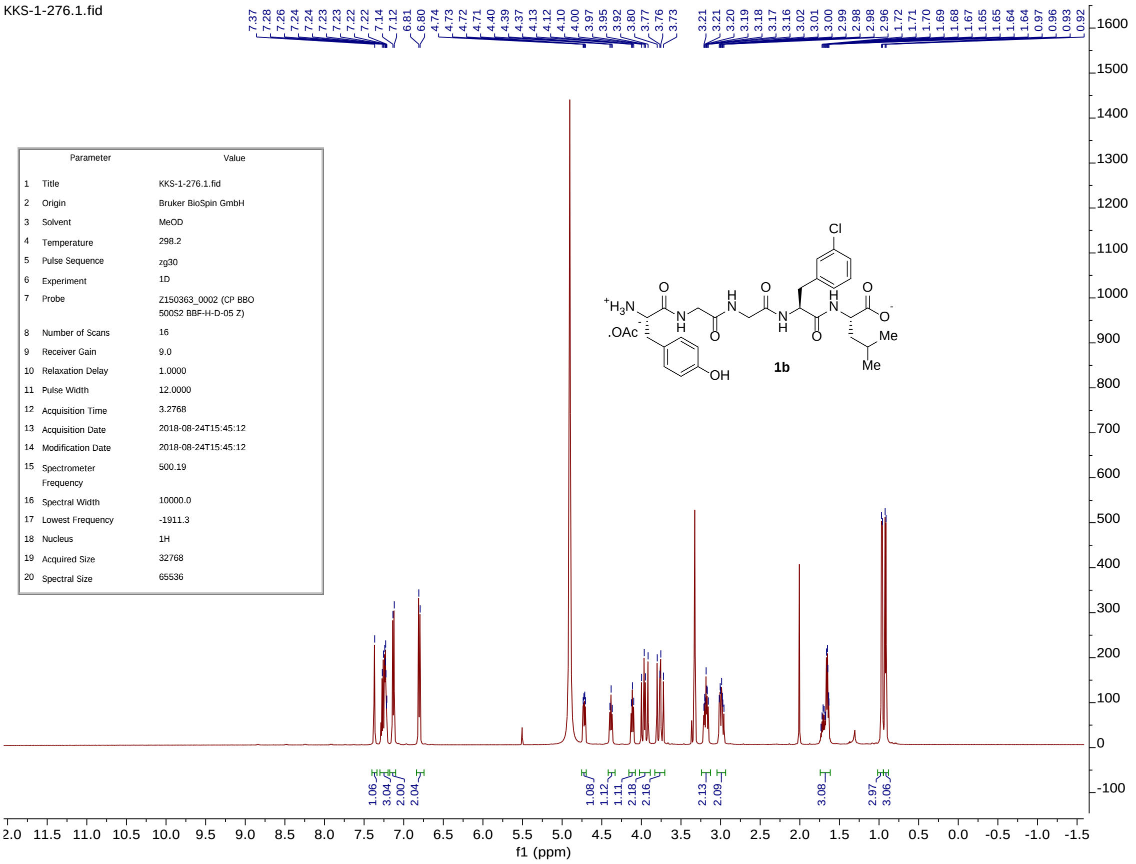
KKS-1-276.1.fid
7.37 7.28 7.26 7.24 7.24 7.23 7.23 7.22 7.22 7.14 7.12 6.81 6.80 4.74 4.73 4.72 4.71 4.40 4.39 4.37 4.13 4.12 4.10 4.00 3.97 3.95 3.92 3.80 3.77 3.76 3.73 3.21 3.21 3.20 3.19 3.18 3.17 3.16 3.02 3.01 3.00 2.99 2.98 2.98 2.96 1.72 1.71 1.70 1.69 1.68 1.67 1.65 1.65 1.64 0.97 0.96 0.93 0.92
Parameter
Value
1 Title KKS-1-276.1.fid
2 Origin Bruker BioSpin GmbH
3 Solvent MeOD
4 Temperature 298.2
5 Pulse Sequence zg30
6 Experiment 1D
7 Probe Z150363_0002 (CP BBO 500S2 BBF-H-D-05 Z)
8 Number of Scans 16
9 Receiver Gain 9.0
10 Relaxation Delay 1.0000
11 Pulse Width 12.0000
12 Acquisition Time 3.2768
13 Acquisition Date 2018-08-24T15:45:12
14 Modification Date 2018-08-24T15:45:12
15 Spectrometer Frequency 500.19
16 Spectral Width 10000.0
17 Lowest Frequency -1911.3
18 Nucleus 1H
19 Acquired Size 32768
20 Spectral Size 65536
Cl
+H3N
.OAc
OH
Me
Me
O-
1b
1.06 3.04 2.00 2.04 1.08 1.12 1.11 2.18 2.16 2.13 2.09 3.08 2.97 3.06
f1 (ppm)

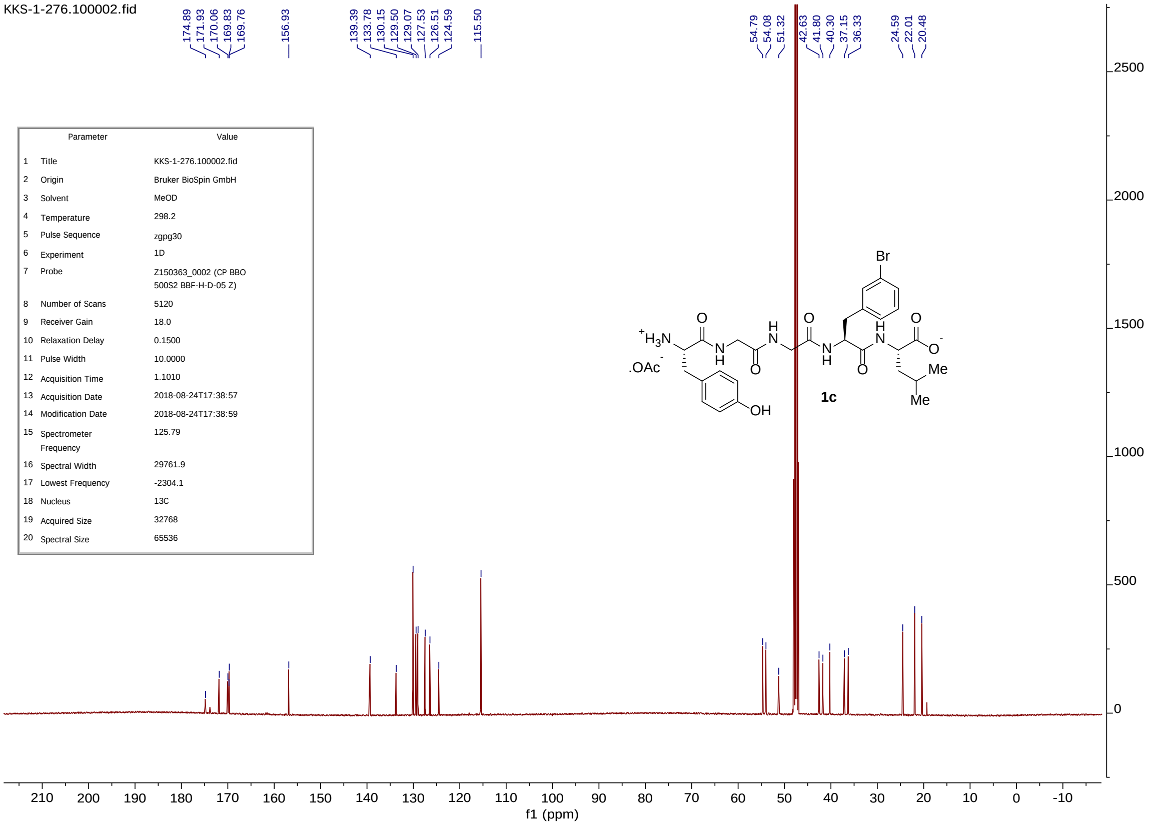

Parameter		Value
1	Title	KKS-1-276.100002.fid
2	Origin	Bruker BioSpin GmbH
3	Solvent	MeOD
4	Temperature	298.2
5	Pulse Sequence	zgpg30
6	Experiment	1D
7	Probe	Z150363_0002 (CP BBO 500S2 BBF-H-D-05 Z)
8	Number of Scans	5120
9	Receiver Gain	18.0
10	Relaxation Delay	0.1500
11	Pulse Width	10.0000
12	Acquisition Time	1.1010
13	Acquisition Date	2018-08-24T17:38:57
14	Modification Date	2018-08-24T17:38:59
15	Spectrometer Frequency	125.79
16	Spectral Width	29761.9
17	Lowest Frequency	-2304.1
18	Nucleus	13C
19	Acquired Size	32768
20	Spectral Size	65536

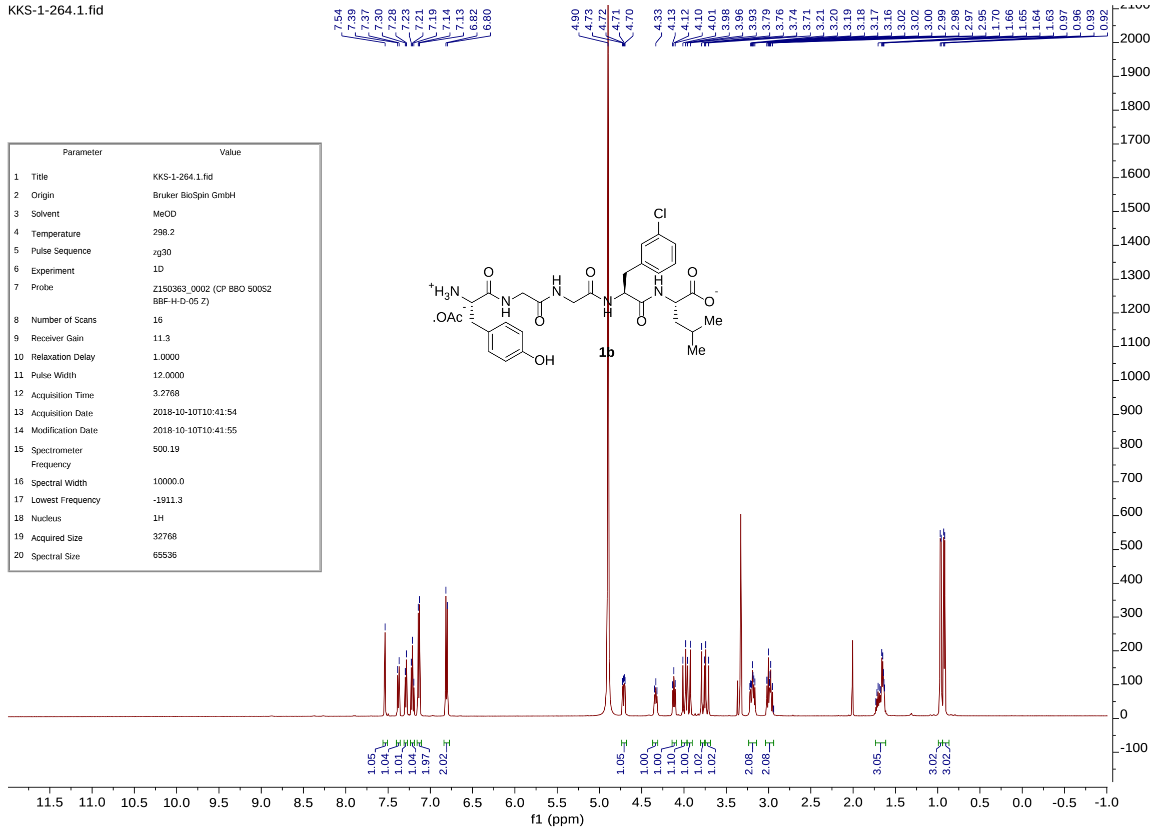

Parameter		Value
1	Title	KKS-1-264.1.fid
2	Origin	Bruker BioSpin GmbH
3	Solvent	MeOD
4	Temperature	298.2
5	Pulse Sequence	zg30
6	Experiment	1D
7	Probe	Z150363_0002 (CP BBO 500S2 BBF-H-D-05 Z)
8	Number of Scans	16
9	Receiver Gain	11.3
10	Relaxation Delay	1.0000
11	Pulse Width	12.0000
12	Acquisition Time	3.2768
13	Acquisition Date	2018-10-10T10:41:54
14	Modification Date	2018-10-10T10:41:55
15	Spectrometer Frequency	500.19
16	Spectral Width	10000.0
17	Lowest Frequency	-1911.3
18	Nucleus	1H
19	Acquired Size	32768
20	Spectral Size	65536

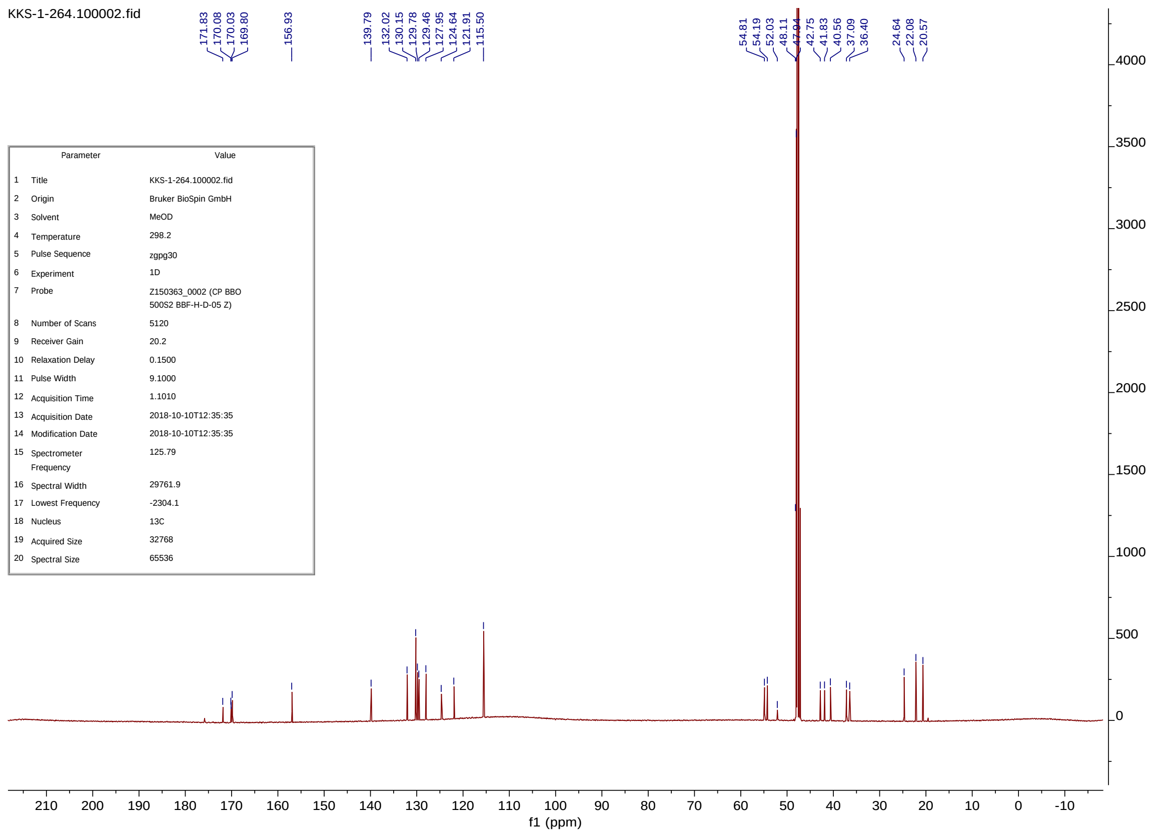

	Parameter	Value
1	Title	KKS-1-264.100002.fid
2	Origin	Bruker BioSpin GmbH
3	Solvent	MeOD
4	Temperature	298.2
5	Pulse Sequence	zgpg30
6	Experiment	1D
7	Probe	Z150363_0002 (CP BBO 500S2 BBF-H-D-05 Z)
8	Number of Scans	5120
9	Receiver Gain	20.2
10	Relaxation Delay	0.1500
11	Pulse Width	9.1000
12	Acquisition Time	1.1010
13	Acquisition Date	2018-10-10T12:35:35
14	Modification Date	2018-10-10T12:35:35
15	Spectrometer Frequency	125.79
16	Spectral Width	29761.9
17	Lowest Frequency	-2304.1
18	Nucleus	13C
19	Acquired Size	32768
20	Spectral Size	65536

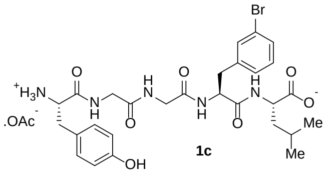

1c

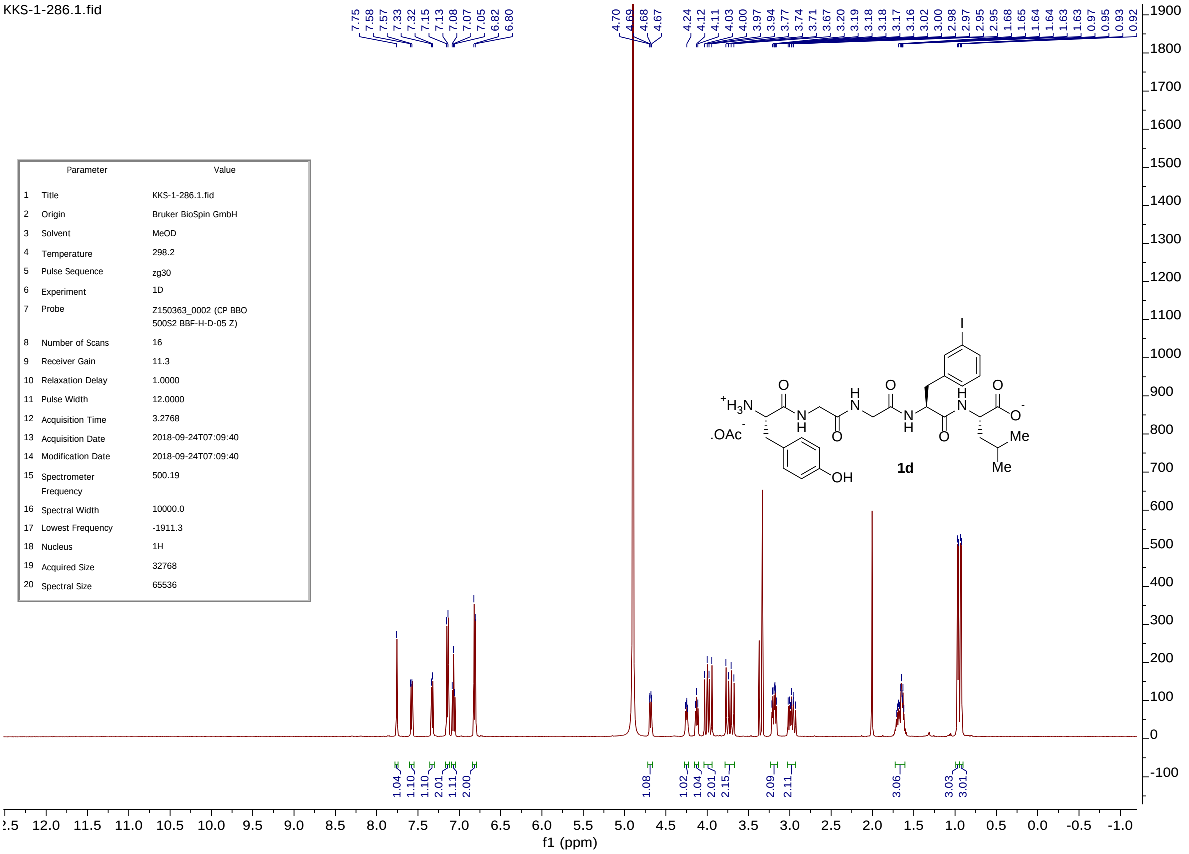

	Parameter	Value
1	Title	KKS-1-286.1.fid
2	Origin	Bruker BioSpin GmbH
3	Solvent	MeOD
4	Temperature	298.2
5	Pulse Sequence	zg30
6	Experiment	1D
7	Probe	Z150363_0002 (CP BBO 500S2 BBF-H-D-05 Z)
8	Number of Scans	16
9	Receiver Gain	11.3
10	Relaxation Delay	1.0000
11	Pulse Width	12.0000
12	Acquisition Time	3.2768
13	Acquisition Date	2018-09-24T07:09:40
14	Modification Date	2018-09-24T07:09:40
15	Spectrometer Frequency	500.19
16	Spectral Width	10000.0
17	Lowest Frequency	-1911.3
18	Nucleus	1H
19	Acquired Size	32768
20	Spectral Size	65536

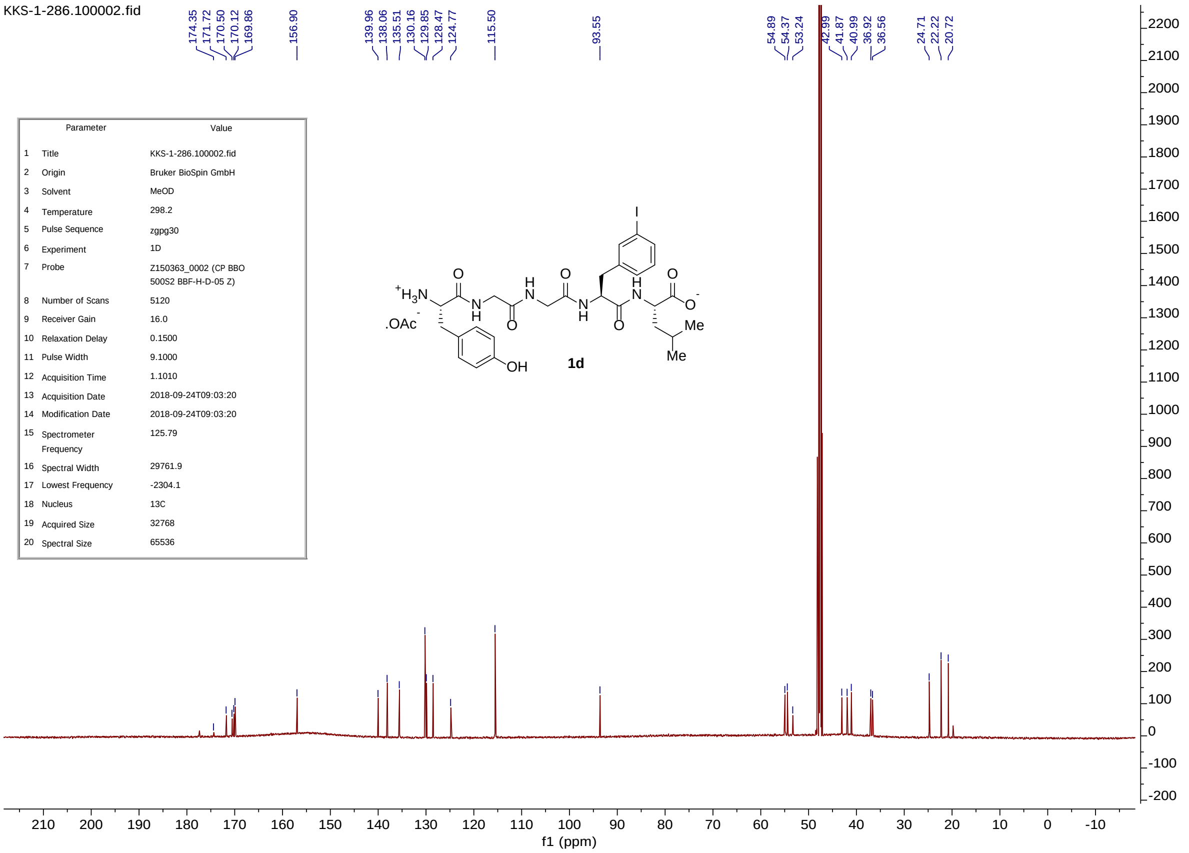

	Parameter	Value
1	Title	KKS-1-286.100002.fid
2	Origin	Bruker BioSpin GmbH
3	Solvent	MeOD
4	Temperature	298.2
5	Pulse Sequence	zgpg30
6	Experiment	1D
7	Probe	Z150363_0002 (CP BBO 500S2 BBF-H-D-05 Z)
8	Number of Scans	5120
9	Receiver Gain	16.0
10	Relaxation Delay	0.1500
11	Pulse Width	9.1000
12	Acquisition Time	1.1010
13	Acquisition Date	2018-09-24T09:03:20
14	Modification Date	2018-09-24T09:03:20
15	Spectrometer Frequency	125.79
16	Spectral Width	29761.9
17	Lowest Frequency	-2304.1
18	Nucleus	13C
19	Acquired Size	32768
20	Spectral Size	65536

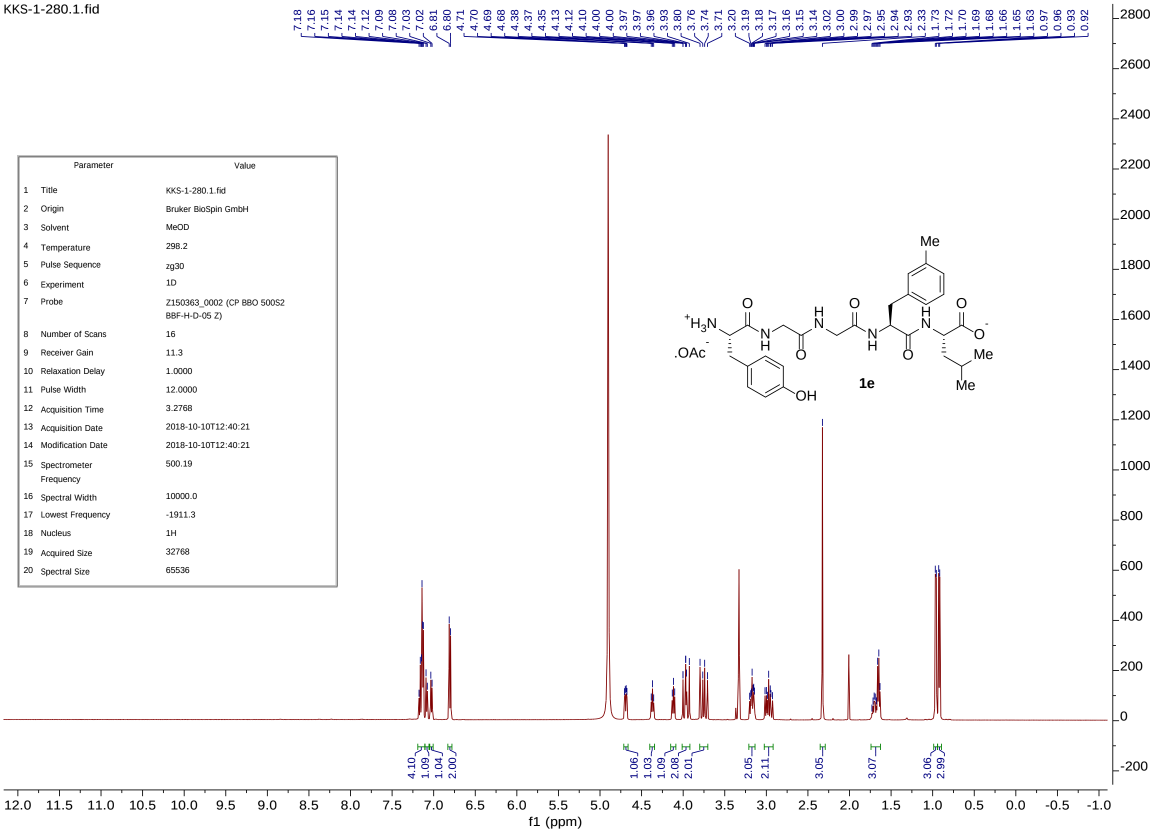

	Parameter	Value
1	Title	KKS-1-280.1.fid
2	Origin	Bruker BioSpin GmbH
3	Solvent	MeOD
4	Temperature	298.2
5	Pulse Sequence	zg30
6	Experiment	1D
7	Probe	Z150363_0002 (CP BBO 500S2 BBF-H-D-05 Z)
8	Number of Scans	16
9	Receiver Gain	11.3
10	Relaxation Delay	1.0000
11	Pulse Width	12.0000
12	Acquisition Time	3.2768
13	Acquisition Date	2018-10-10T12:40:21
14	Modification Date	2018-10-10T12:40:21
15	Spectrometer Frequency	500.19
16	Spectral Width	10000.0
17	Lowest Frequency	-1911.3
18	Nucleus	1H
19	Acquired Size	32768
20	Spectral Size	65536

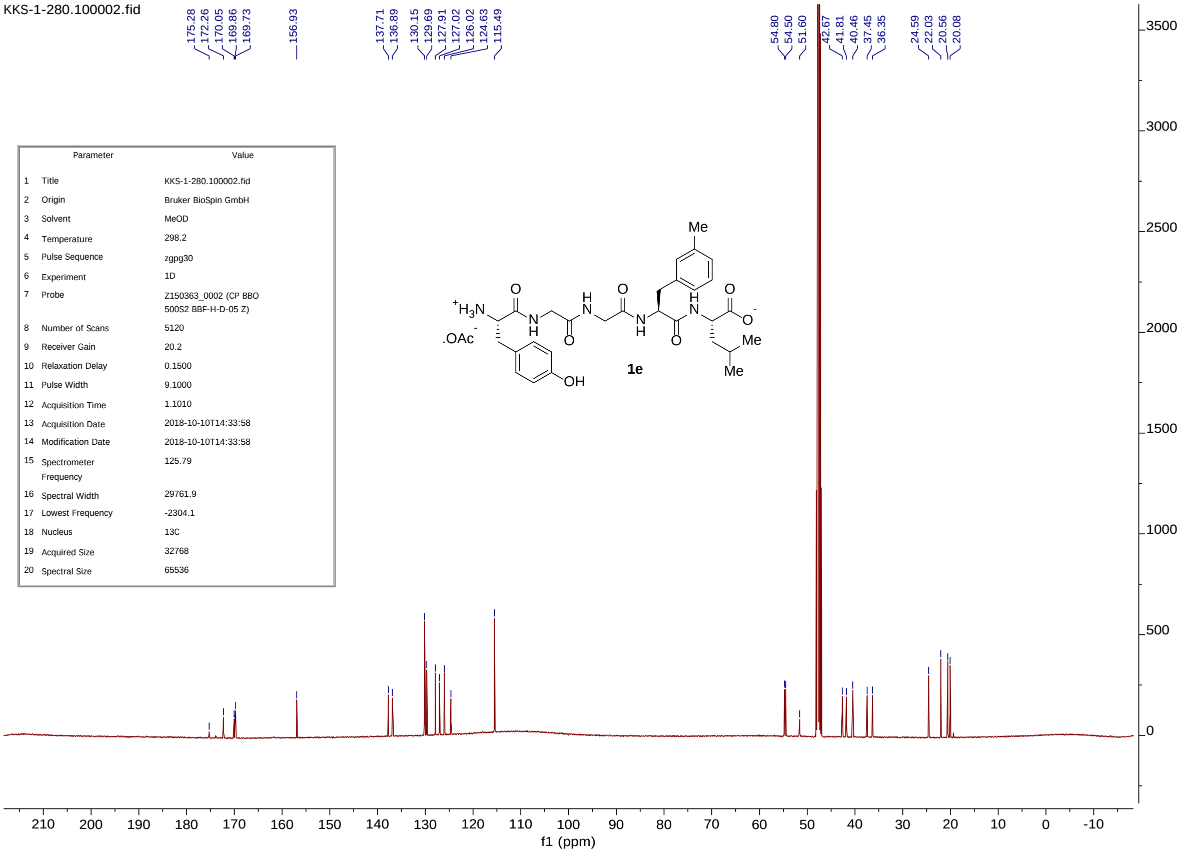

	Parameter	Value
1	Title	KKS-1-280.100002.fid
2	Origin	Bruker BioSpin GmbH
3	Solvent	MeOD
4	Temperature	298.2
5	Pulse Sequence	zgpg30
6	Experiment	1D
7	Probe	Z150363_0002 (CP BBO 500S2 BBF-H-D-05 Z)
8	Number of Scans	5120
9	Receiver Gain	20.2
10	Relaxation Delay	0.1500
11	Pulse Width	9.1000
12	Acquisition Time	1.1010
13	Acquisition Date	2018-10-10T14:33:58
14	Modification Date	2018-10-10T14:33:58
15	Spectrometer Frequency	125.79
16	Spectral Width	29761.9
17	Lowest Frequency	-2304.1
18	Nucleus	13C
19	Acquired Size	32768
20	Spectral Size	65536

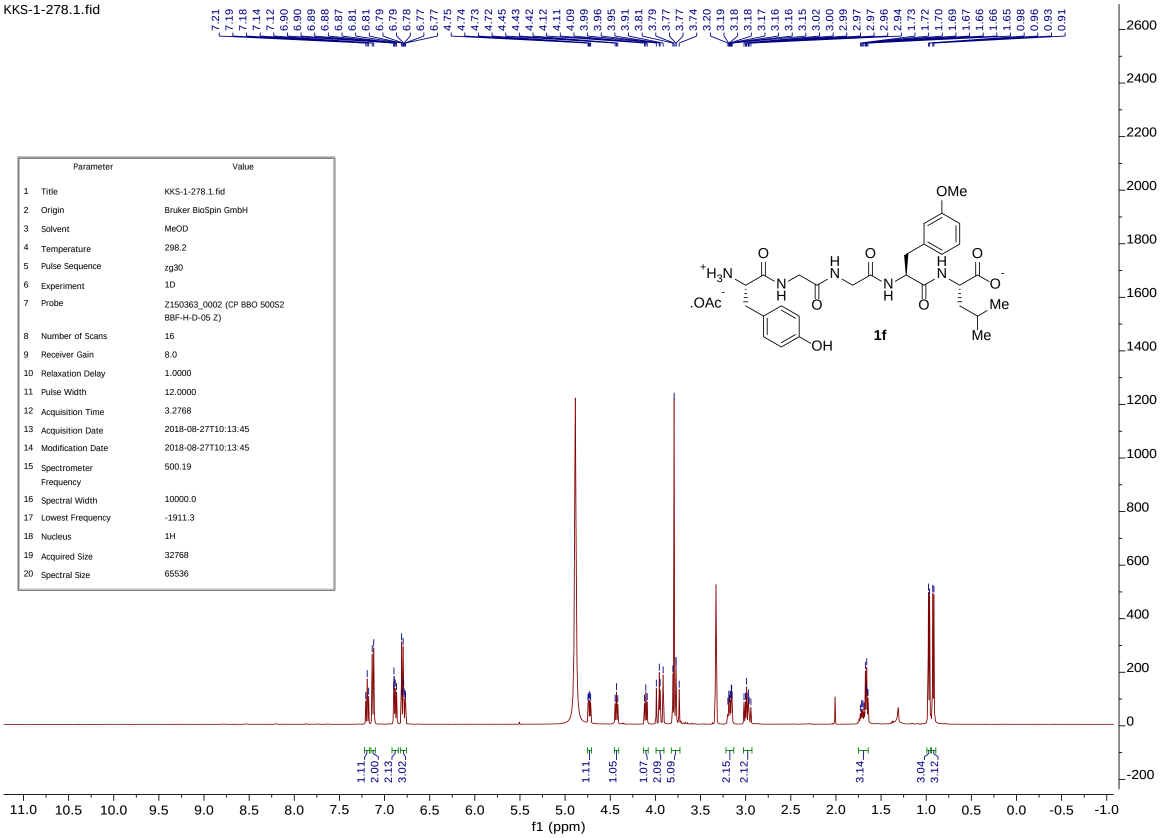

	Parameter	Value
1	Title	KKS-1-278.1.fid
2	Origin	Bruker BioSpin GmbH
3	Solvent	MeOD
4	Temperature	298.2
5	Pulse Sequence	zg30
6	Experiment	1D
7	Probe	Z150363_0002 (CP BBO 500S2 BBF-H-D-05 Z)
8	Number of Scans	16
9	Receiver Gain	8.0
10	Relaxation Delay	1.0000
11	Pulse Width	12.0000
12	Acquisition Time	3.2768
13	Acquisition Date	2018-08-27T10:13:45
14	Modification Date	2018-08-27T10:13:45
15	Spectrometer Frequency	500.19
16	Spectral Width	10000.0
17	Lowest Frequency	-1911.3
18	Nucleus	1H
19	Acquired Size	32768
20	Spectral Size	65536

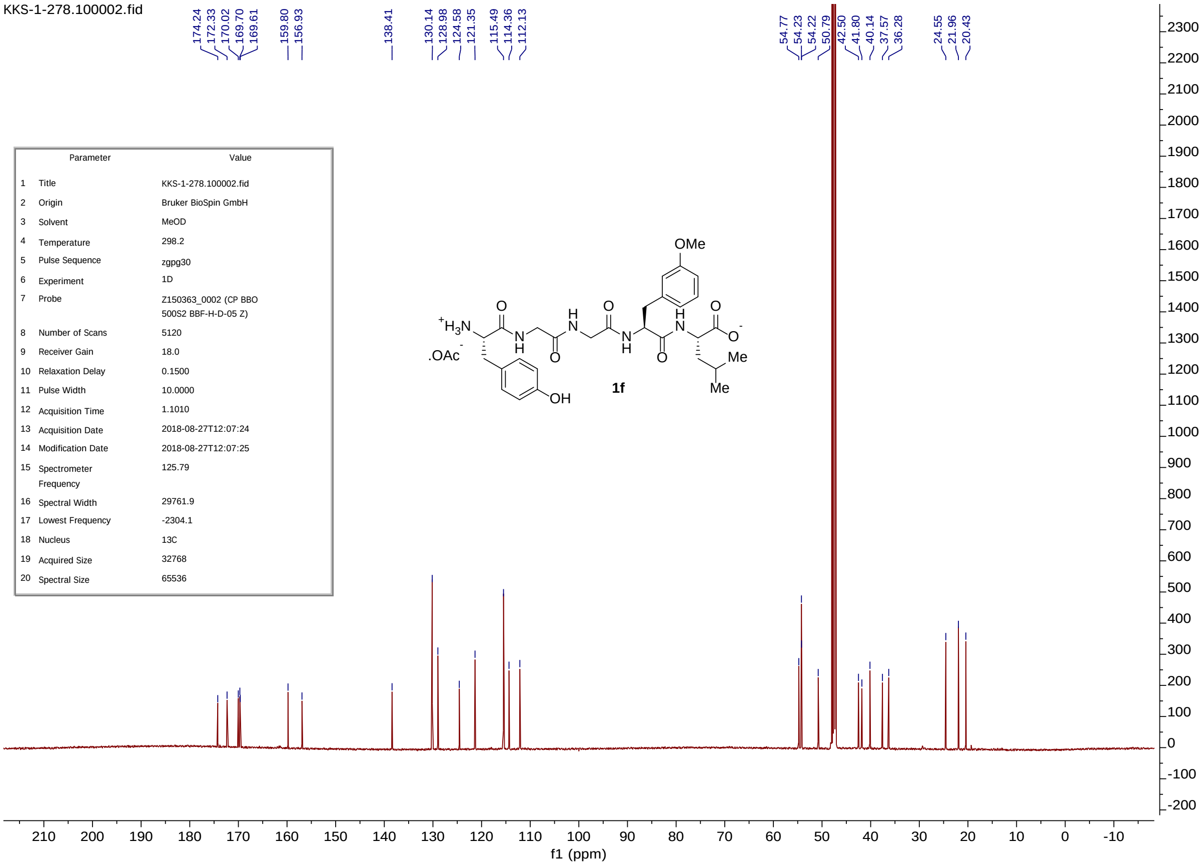

	Parameter	Value
1	Title	KKS-1-278.100002.fid
2	Origin	Bruker BioSpin GmbH
3	Solvent	MeOD
4	Temperature	298.2
5	Pulse Sequence	zgpg30
6	Experiment	1D
7	Probe	Z150363_0002 (CP BBO 500S2 BBF-H-D-05 Z)
8	Number of Scans	5120
9	Receiver Gain	18.0
10	Relaxation Delay	0.1500
11	Pulse Width	10.0000
12	Acquisition Time	1.1010
13	Acquisition Date	2018-08-27T12:07:24
14	Modification Date	2018-08-27T12:07:25
15	Spectrometer Frequency	125.79
16	Spectral Width	29761.9
17	Lowest Frequency	-2304.1
18	Nucleus	13C
19	Acquired Size	32768
20	Spectral Size	65536

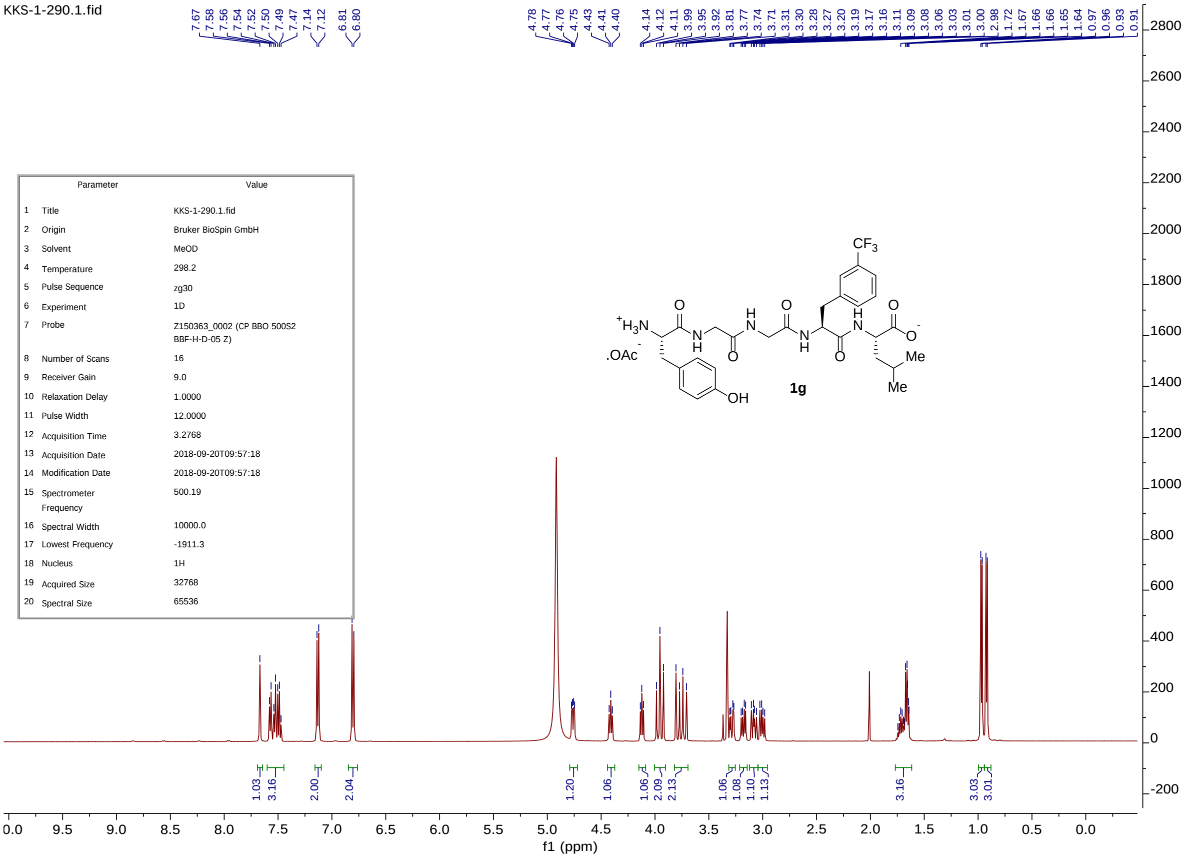

	Parameter	Value
1	Title	KKS-1-290.1.fid
2	Origin	Bruker BioSpin GmbH
3	Solvent	MeOD
4	Temperature	298.2
5	Pulse Sequence	zg30
6	Experiment	1D
7	Probe	Z150363_0002 (CP BBO 500S2 BBF-H-D-05 Z)
8	Number of Scans	16
9	Receiver Gain	9.0
10	Relaxation Delay	1.0000
11	Pulse Width	12.0000
12	Acquisition Time	3.2768
13	Acquisition Date	2018-09-20T09:57:18
14	Modification Date	2018-09-20T09:57:18
15	Spectrometer Frequency	500.19
16	Spectral Width	10000.0
17	Lowest Frequency	-1911.3
18	Nucleus	1H
19	Acquired Size	32768
20	Spectral Size	65536

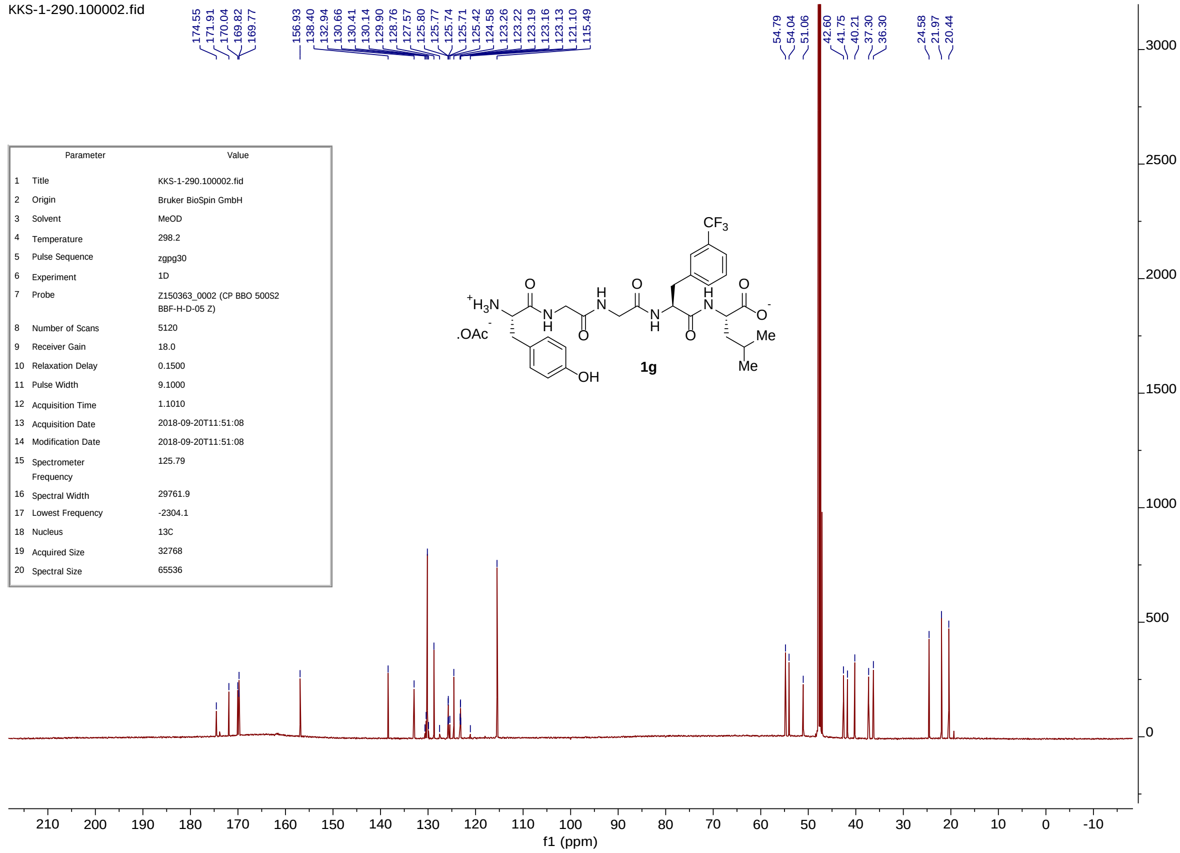

	Parameter	Value
1	Title	KKS-1-290.100002.fid
2	Origin	Bruker BioSpin GmbH
3	Solvent	MeOD
4	Temperature	298.2
5	Pulse Sequence	zgpg30
6	Experiment	1D
7	Probe	Z150363_0002 (CP BBO 500S2 BBF-H-D-05 Z)
8	Number of Scans	5120
9	Receiver Gain	18.0
10	Relaxation Delay	0.1500
11	Pulse Width	9.1000
12	Acquisition Time	1.1010
13	Acquisition Date	2018-09-20T11:51:08
14	Modification Date	2018-09-20T11:51:08
15	Spectrometer Frequency	125.79
16	Spectral Width	29761.9
17	Lowest Frequency	-2304.1
18	Nucleus	13C
19	Acquired Size	32768
20	Spectral Size	65536

kks-1-290_fr4.1.fid

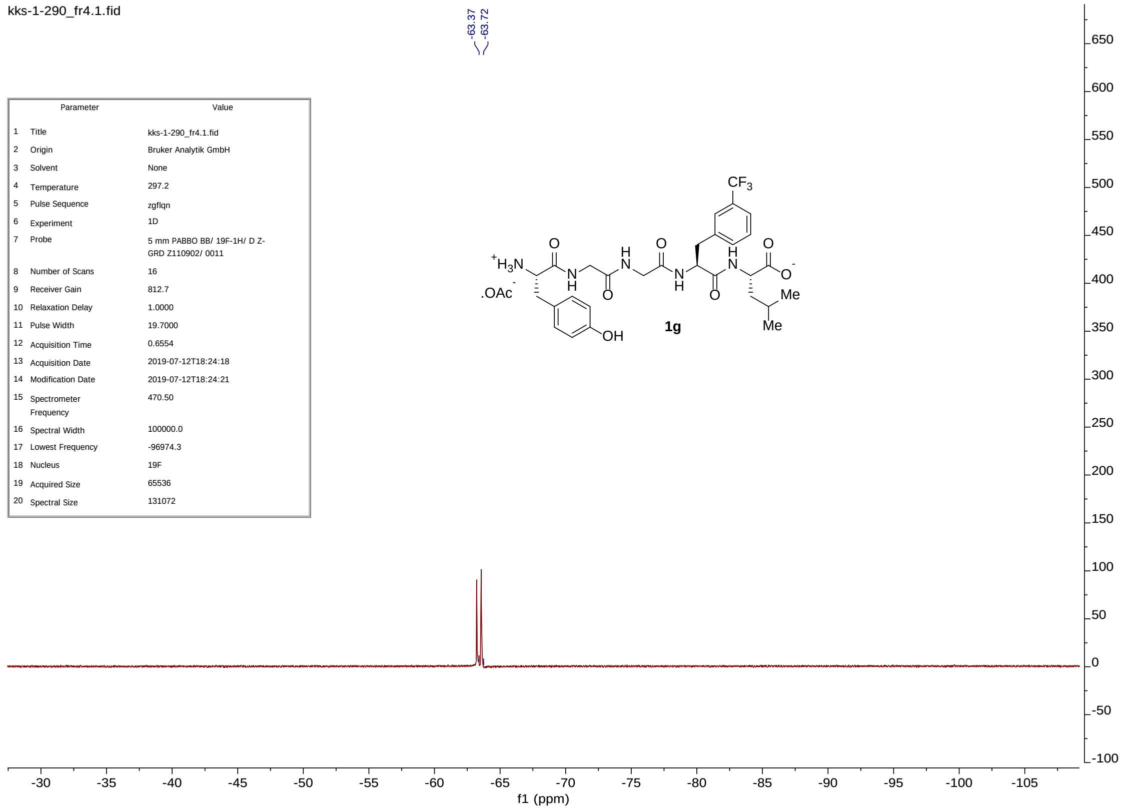

1g

	Parameter	Value
1	Title	kks-1-290_fr4.1.fid
2	Origin	Bruker Analytik GmbH
3	Solvent	None
4	Temperature	297.2
5	Pulse Sequence	zgflqn
6	Experiment	1D
7	Probe	5 mm PABBO BB/ 19F-1H/ D Z-GRD Z110902/ 0011
8	Number of Scans	16
9	Receiver Gain	812.7
10	Relaxation Delay	1.0000
11	Pulse Width	19.7000
12	Acquisition Time	0.6554
13	Acquisition Date	2019-07-12T18:24:18
14	Modification Date	2019-07-12T18:24:21
15	Spectrometer Frequency	470.50
16	Spectral Width	100000.0
17	Lowest Frequency	-96974.3
18	Nucleus	19F
19	Acquired Size	65536
20	Spectral Size	131072

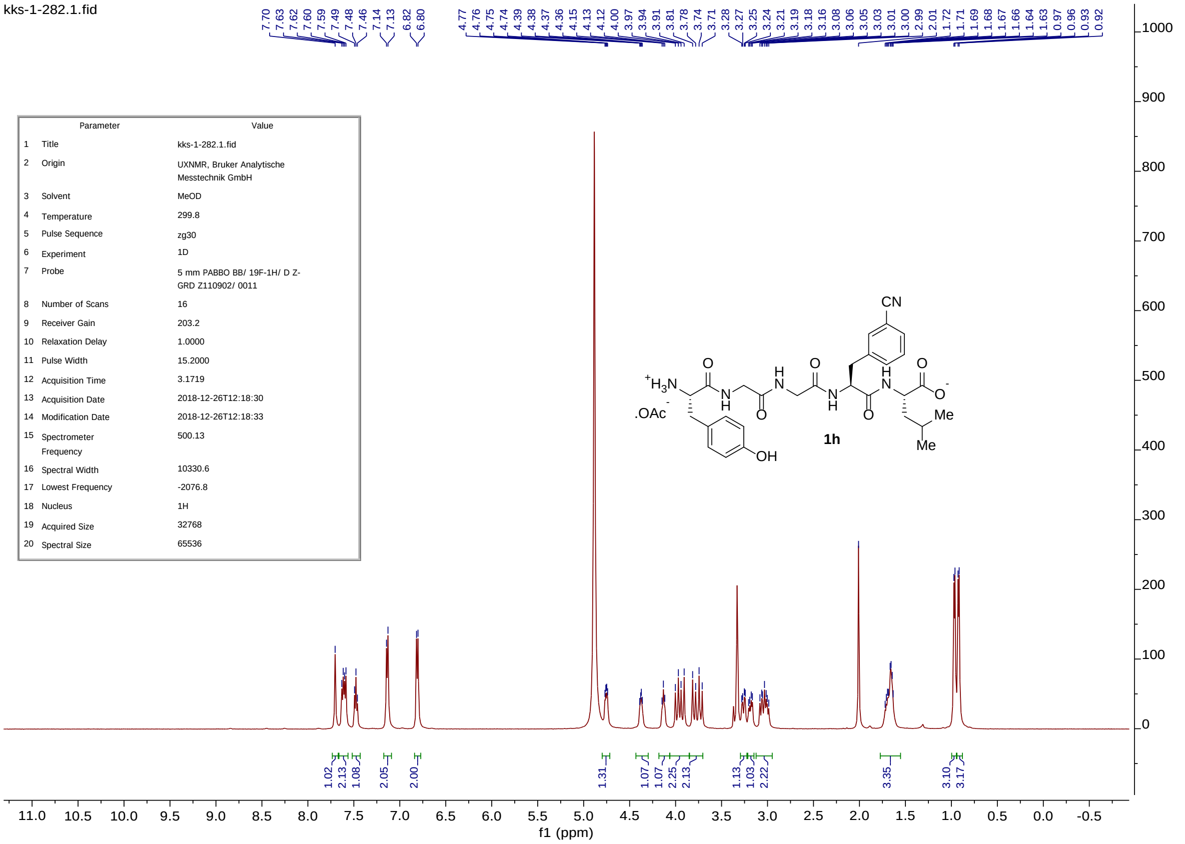

	Parameter	Value
1	Title	kks-1-282.1.fid
2	Origin	UXNMR, Bruker Analytische Messtechnik GmbH
3	Solvent	MeOD
4	Temperature	299.8
5	Pulse Sequence	zg30
6	Experiment	1D
7	Probe	5 mm PABBO BB/ 19F-1H/ D Z-GRD Z110902/ 0011
8	Number of Scans	16
9	Receiver Gain	203.2
10	Relaxation Delay	1.0000
11	Pulse Width	15.2000
12	Acquisition Time	3.1719
13	Acquisition Date	2018-12-26T12:18:30
14	Modification Date	2018-12-26T12:18:33
15	Spectrometer Frequency	500.13
16	Spectral Width	10330.6
17	Lowest Frequency	-2076.8
18	Nucleus	1H
19	Acquired Size	32768
20	Spectral Size	65536

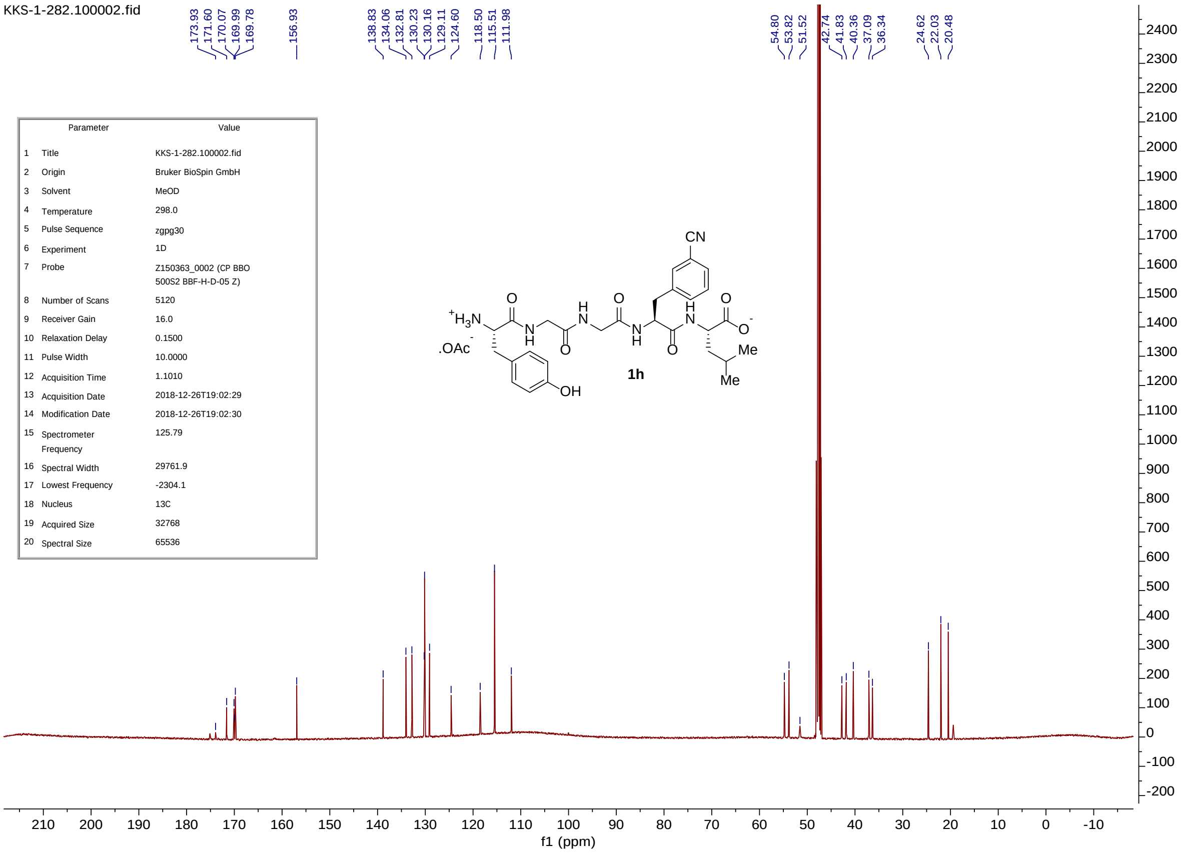

	Parameter	Value
1	Title	KKS-1-282.100002.fid
2	Origin	Bruker BioSpin GmbH
3	Solvent	MeOD
4	Temperature	298.0
5	Pulse Sequence	zgpg30
6	Experiment	1D
7	Probe	Z150363_0002 (CP BBO 500S2 BBF-H-D-05 Z)
8	Number of Scans	5120
9	Receiver Gain	16.0
10	Relaxation Delay	0.1500
11	Pulse Width	10.0000
12	Acquisition Time	1.1010
13	Acquisition Date	2018-12-26T19:02:29
14	Modification Date	2018-12-26T19:02:30
15	Spectrometer Frequency	125.79
16	Spectral Width	29761.9
17	Lowest Frequency	-2304.1
18	Nucleus	13C
19	Acquired Size	32768
20	Spectral Size	65536

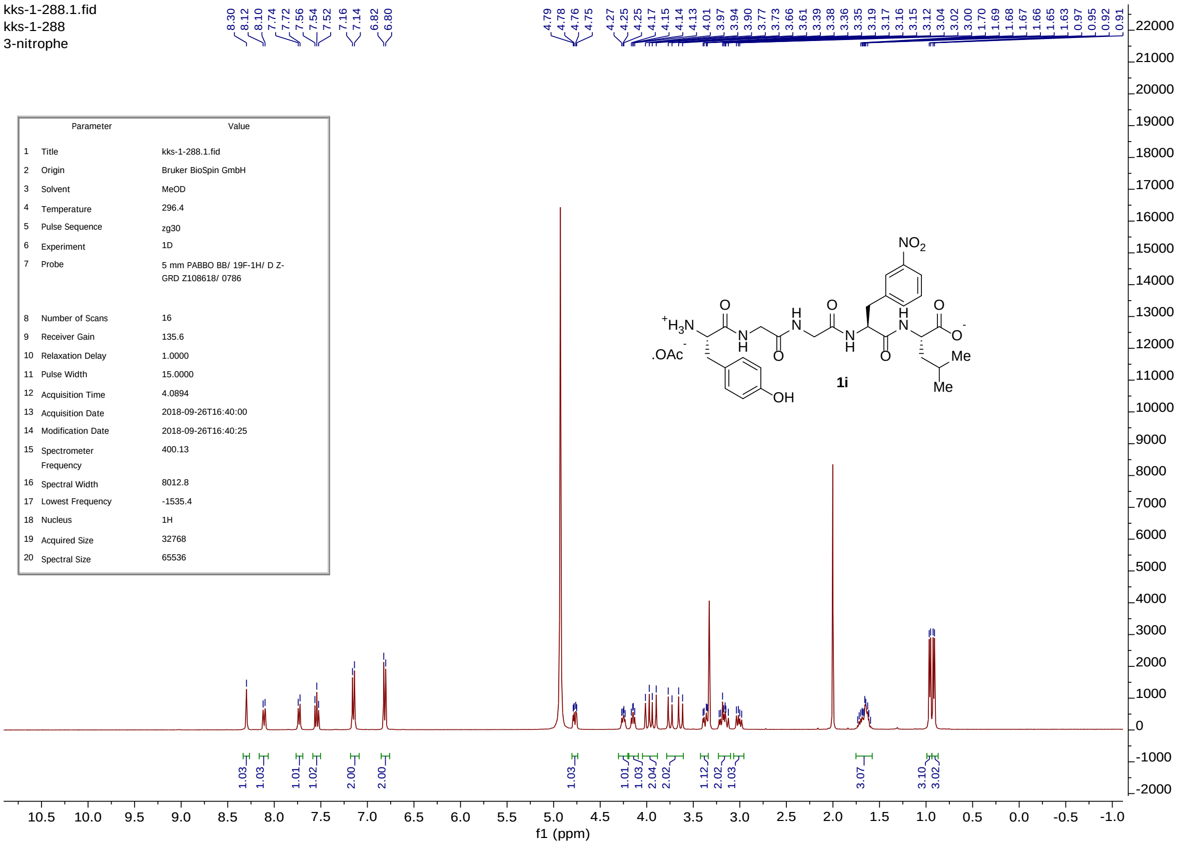

kks-1-288.1.fid
kks-1-288
3-nitrophe
8.30 8.12 8.10 7.74 7.72 7.56 7.54 7.52 7.16 7.14 6.82 6.80
4.79 4.78 4.76 4.75 4.27 4.25 4.25 4.17 4.15 4.14 4.13 4.01 3.97 3.94 3.90 3.77 3.73 3.66 3.61 3.39 3.38 3.36 3.35 3.19 3.17 3.16 3.15 3.12 3.04 3.02 3.00 1.70 1.69 1.68 1.67 1.66 1.65 1.63 0.97 0.95 0.92 0.91
Parameter Value
1 Title kks-1-288.1.fid
2 Origin Bruker BioSpin GmbH
3 Solvent MeOD
4 Temperature 296.4
5 Pulse Sequence zg30
6 Experiment 1D
7 Probe 5 mm PABBO BB/ 19F-1H/ D Z-GRD Z108618/ 0786
8 Number of Scans 16
9 Receiver Gain 135.6
10 Relaxation Delay 1.0000
11 Pulse Width 15.0000
12 Acquisition Time 4.0894
13 Acquisition Date 2018-09-26T16:40:00
14 Modification Date 2018-09-26T16:40:25
15 Spectrometer Frequency 400.13
16 Spectral Width 8012.8
17 Lowest Frequency -1535.4
18 Nucleus 1H
19 Acquired Size 32768
20 Spectral Size 65536
NO2
+H3N
.OAc-
OH
1i
Me
Me
1.03 1.03 1.01 1.02 2.00 2.00 1.03 1.01 1.03 2.04 2.02 1.12 2.02 1.03 3.07 3.10 3.02
f1 (ppm)

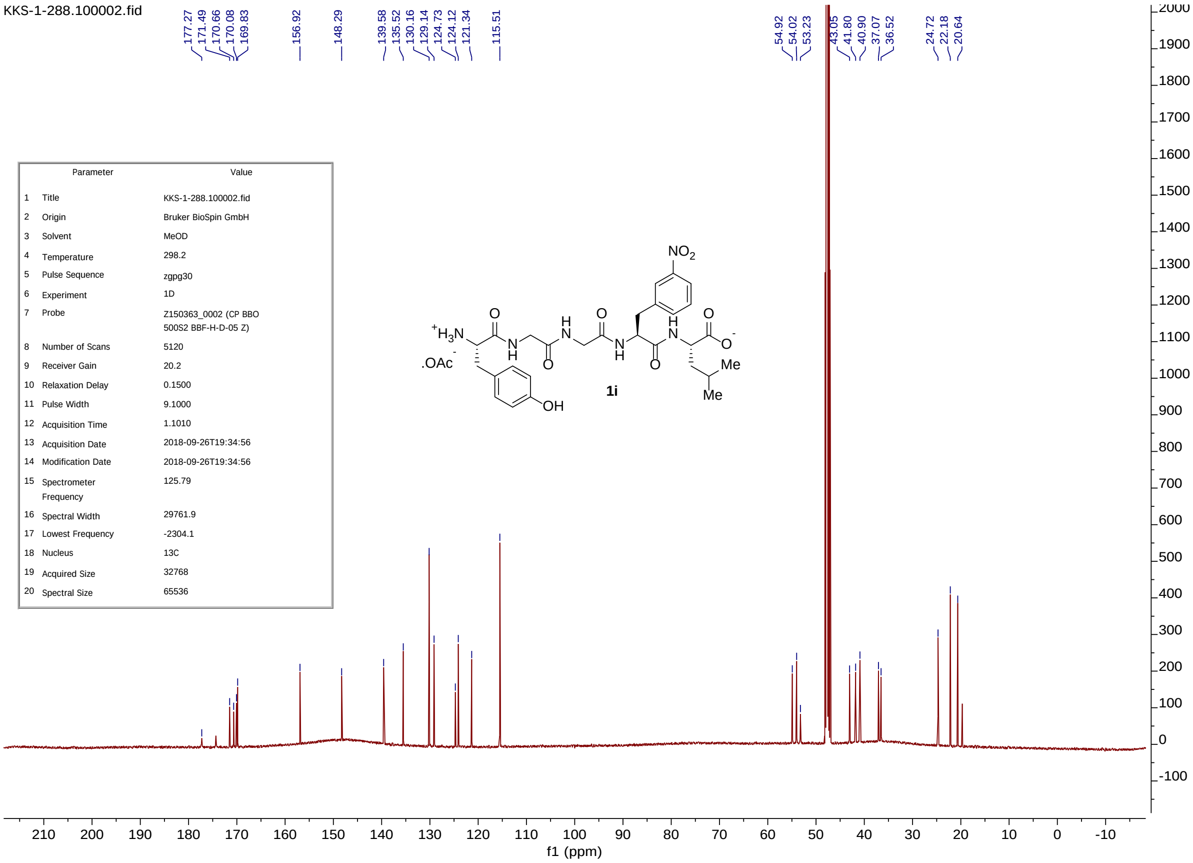

KKS-1-288.100002.fid
177.27 171.49 170.66 170.08 169.83 156.92 148.29 139.58 135.52 130.16 129.14 124.73 124.12 121.34 115.51
54.92 54.02 53.23 43.05 41.80 40.90 37.07 36.52 24.72 22.18 20.64
Parameter Value
1 Title KKS-1-288.100002.fid
2 Origin Bruker BioSpin GmbH
3 Solvent MeOD
4 Temperature 298.2
5 Pulse Sequence zgpg30
6 Experiment 1D
7 Probe Z150363_0002 (CP BBO 500S2 BBF-H-D-05 Z)
8 Number of Scans 5120
9 Receiver Gain 20.2
10 Relaxation Delay 0.1500
11 Pulse Width 9.1000
12 Acquisition Time 1.1010
13 Acquisition Date 2018-09-26T19:34:56
14 Modification Date 2018-09-26T19:34:56
15 Spectrometer Frequency 125.79
16 Spectral Width 29761.9
17 Lowest Frequency -2304.1
18 Nucleus 13C
19 Acquired Size 32768
20 Spectral Size 65536
NO2
+H3N
.OAc-
OH
1i
Me
Me
f1 (ppm)

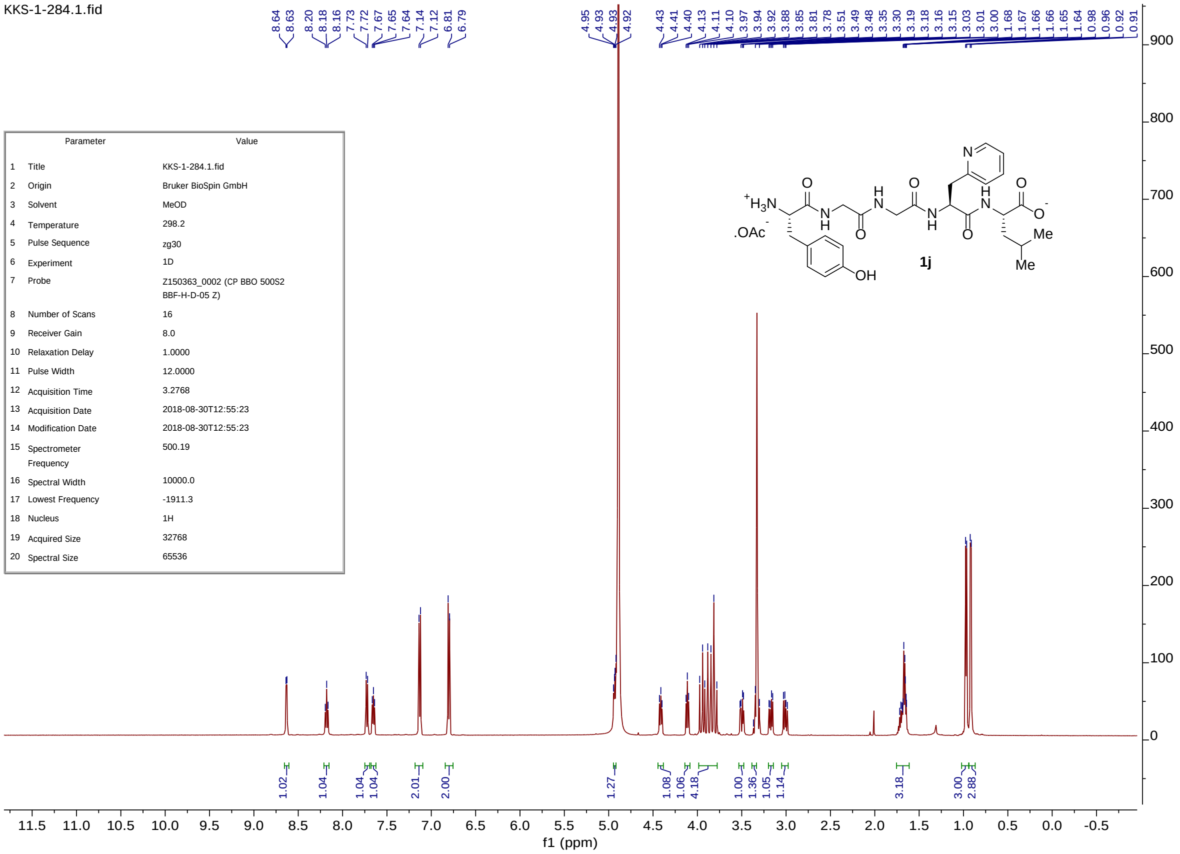

	Parameter	Value
1	Title	KKS-1-284.1.fid
2	Origin	Bruker BioSpin GmbH
3	Solvent	MeOD
4	Temperature	298.2
5	Pulse Sequence	zg30
6	Experiment	1D
7	Probe	Z150363_0002 (CP BBO 500S2 BBF-H-D-05 Z)
8	Number of Scans	16
9	Receiver Gain	8.0
10	Relaxation Delay	1.0000
11	Pulse Width	12.0000
12	Acquisition Time	3.2768
13	Acquisition Date	2018-08-30T12:55:23
14	Modification Date	2018-08-30T12:55:23
15	Spectrometer Frequency	500.19
16	Spectral Width	10000.0
17	Lowest Frequency	-1911.3
18	Nucleus	1H
19	Acquired Size	32768
20	Spectral Size	65536

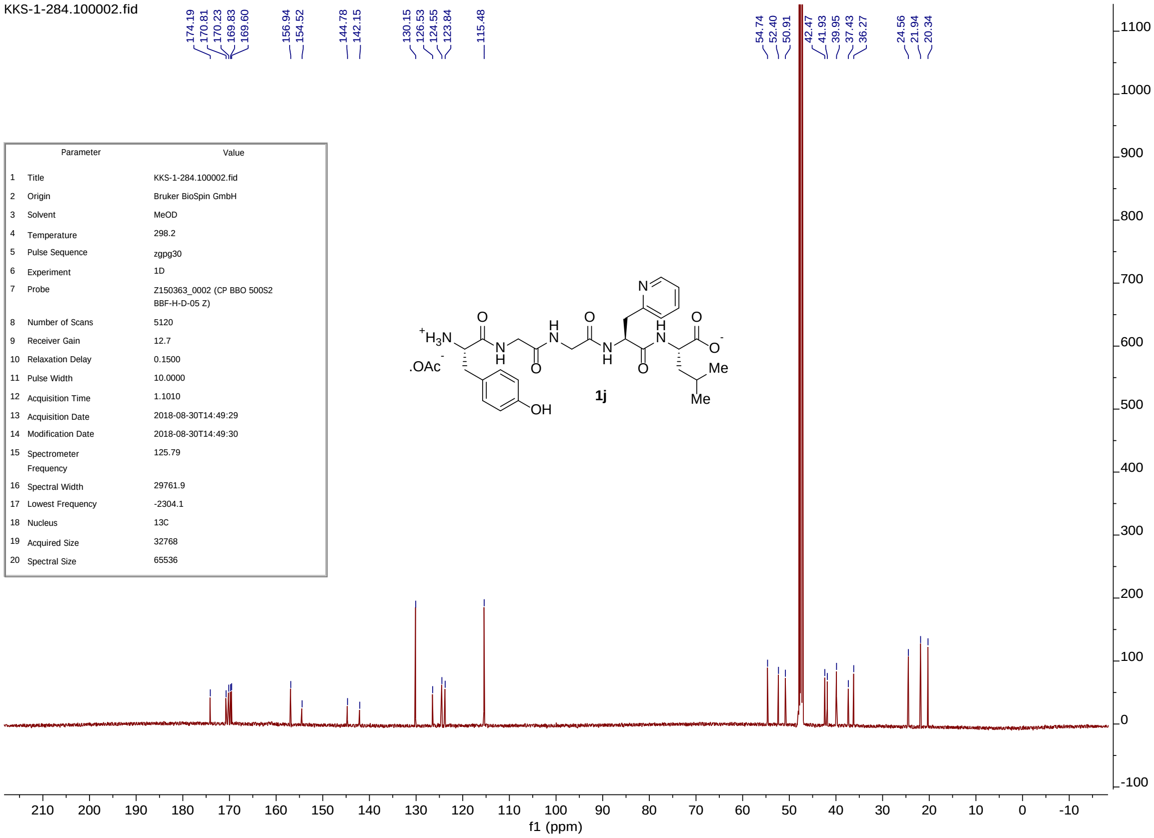

	Parameter	Value
1	Title	KKS-1-284.100002.fid
2	Origin	Bruker BioSpin GmbH
3	Solvent	MeOD
4	Temperature	298.2
5	Pulse Sequence	zgpg30
6	Experiment	1D
7	Probe	Z150363_0002 (CP BBO 500S2 BBF-H-D-05 Z)
8	Number of Scans	5120
9	Receiver Gain	12.7
10	Relaxation Delay	0.1500
11	Pulse Width	10.0000
12	Acquisition Time	1.1010
13	Acquisition Date	2018-08-30T14:49:29
14	Modification Date	2018-08-30T14:49:30
15	Spectrometer Frequency	125.79
16	Spectral Width	29761.9
17	Lowest Frequency	-2304.1
18	Nucleus	13C
19	Acquired Size	32768
20	Spectral Size	65536

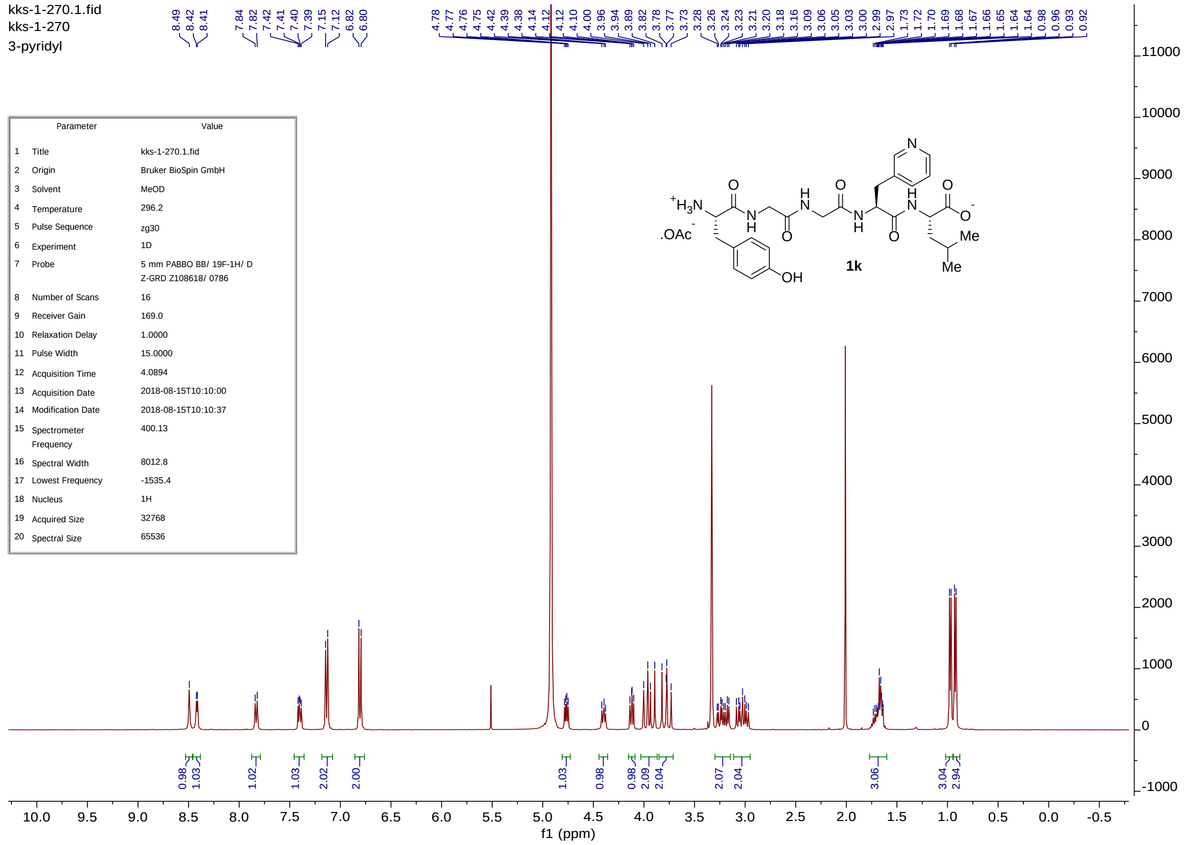

	Parameter	Value
1	Title	kks-1-270.1.fid
2	Origin	Bruker BioSpin GmbH
3	Solvent	MeOD
4	Temperature	296.2
5	Pulse Sequence	zg30
6	Experiment	1D
7	Probe	5 mm PABBO BB/ 19F-1H/ D Z-GRD Z108618/ 0786
8	Number of Scans	16
9	Receiver Gain	169.0
10	Relaxation Delay	1.0000
11	Pulse Width	15.0000
12	Acquisition Time	4.0894
13	Acquisition Date	2018-08-15T10:10:00
14	Modification Date	2018-08-15T10:10:37
15	Spectrometer Frequency	400.13
16	Spectral Width	8012.8
17	Lowest Frequency	-1535.4
18	Nucleus	1H
19	Acquired Size	32768
20	Spectral Size	65536

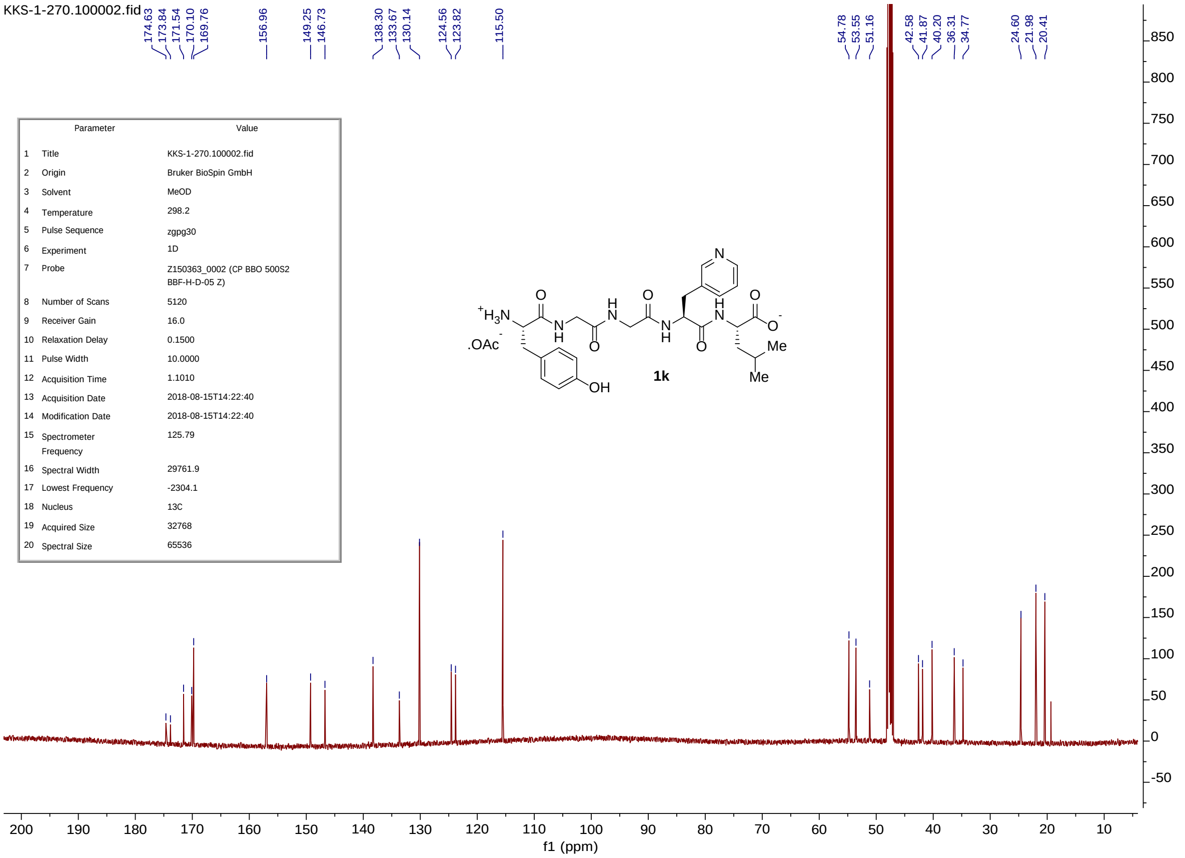

	Parameter	Value
1	Title	KKS-1-270.100002.fid
2	Origin	Bruker BioSpin GmbH
3	Solvent	MeOD
4	Temperature	298.2
5	Pulse Sequence	zgpg30
6	Experiment	1D
7	Probe	Z150363_0002 (CP BBO 500S2 BBF-H-D-05 Z)
8	Number of Scans	5120
9	Receiver Gain	16.0
10	Relaxation Delay	0.1500
11	Pulse Width	10.0000
12	Acquisition Time	1.1010
13	Acquisition Date	2018-08-15T14:22:40
14	Modification Date	2018-08-15T14:22:40
15	Spectrometer Frequency	125.79
16	Spectral Width	29761.9
17	Lowest Frequency	-2304.1
18	Nucleus	13C
19	Acquired Size	32768
20	Spectral Size	65536

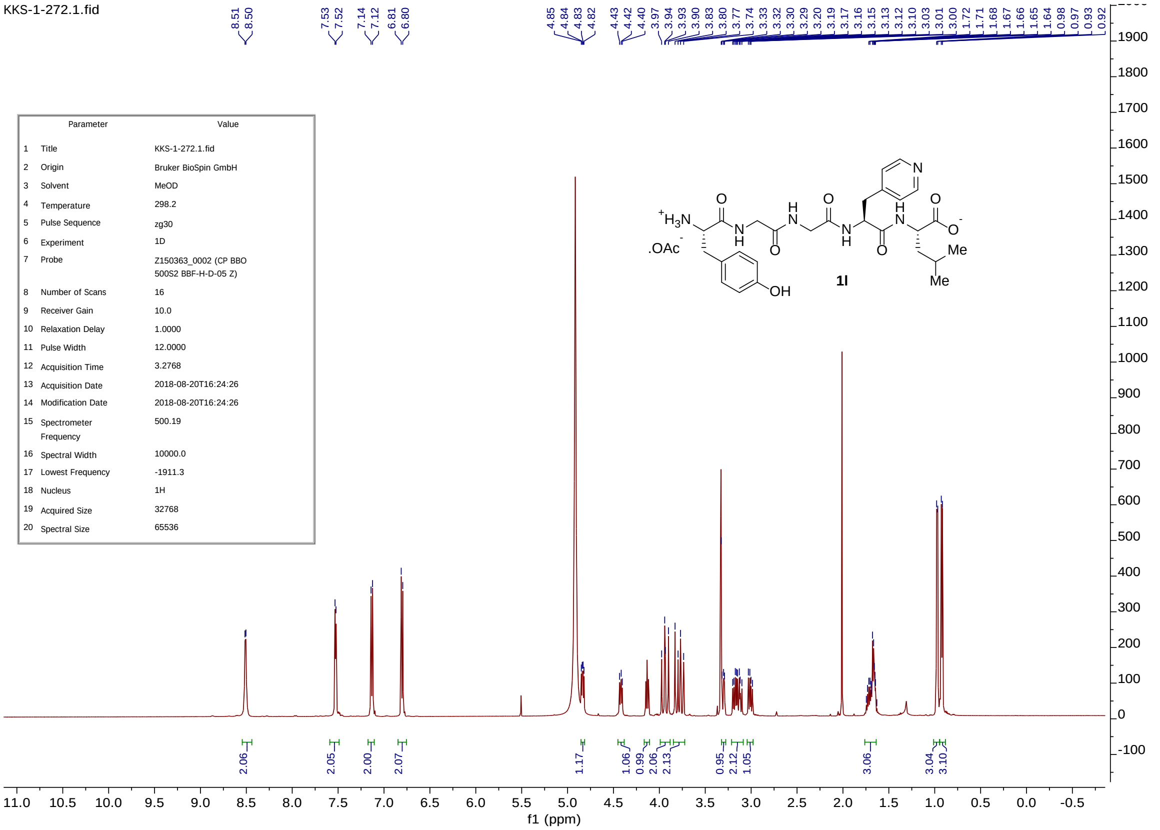

Parameter		Value
1	Title	KKS-1-272.1.fid
2	Origin	Bruker BioSpin GmbH
3	Solvent	MeOD
4	Temperature	298.2
5	Pulse Sequence	zg30
6	Experiment	1D
7	Probe	Z150363_0002 (CP BBO 500S2 BBF-H-D-05 Z)
8	Number of Scans	16
9	Receiver Gain	10.0
10	Relaxation Delay	1.0000
11	Pulse Width	12.0000
12	Acquisition Time	3.2768
13	Acquisition Date	2018-08-20T16:24:26
14	Modification Date	2018-08-20T16:24:26
15	Spectrometer Frequency	500.19
16	Spectral Width	10000.0
17	Lowest Frequency	-1911.3
18	Nucleus	1H
19	Acquired Size	32768
20	Spectral Size	65536

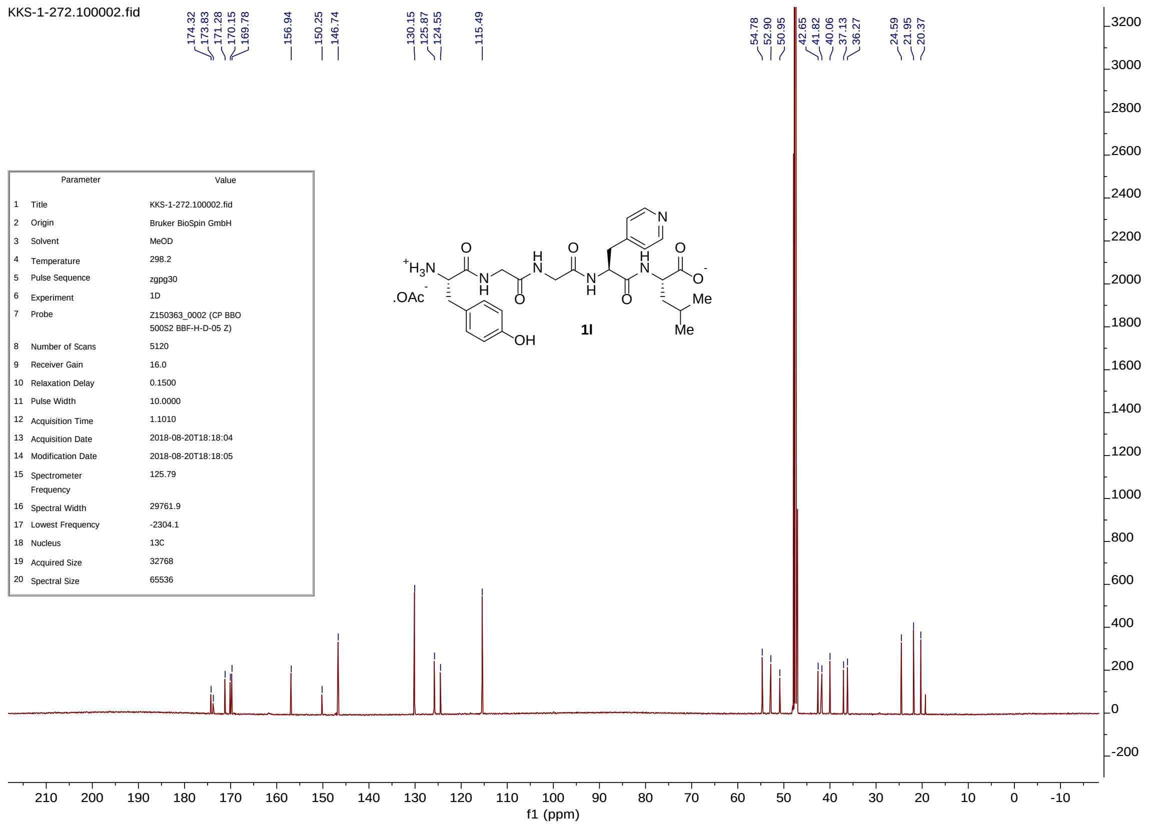

Parameter		Value
1	Title	KKS-1-272.100002.fid
2	Origin	Bruker BioSpin GmbH
3	Solvent	MeOD
4	Temperature	298.2
5	Pulse Sequence	zgpg30
6	Experiment	1D
7	Probe	Z150363_0002 (CP BBO 500S2 BBF-H-D-05 Z)
8	Number of Scans	5120
9	Receiver Gain	16.0
10	Relaxation Delay	0.1500
11	Pulse Width	10.0000
12	Acquisition Time	1.1010
13	Acquisition Date	2018-08-20T18:18:04
14	Modification Date	2018-08-20T18:18:05
15	Spectrometer Frequency	125.79
16	Spectral Width	29761.9
17	Lowest Frequency	-2304.1
18	Nucleus	13C
19	Acquired Size	32768
20	Spectral Size	65536

HPLC Chromatograms of Peptides

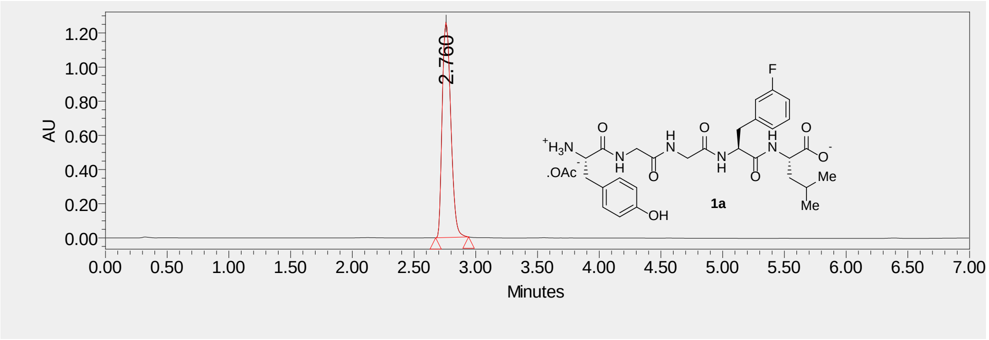

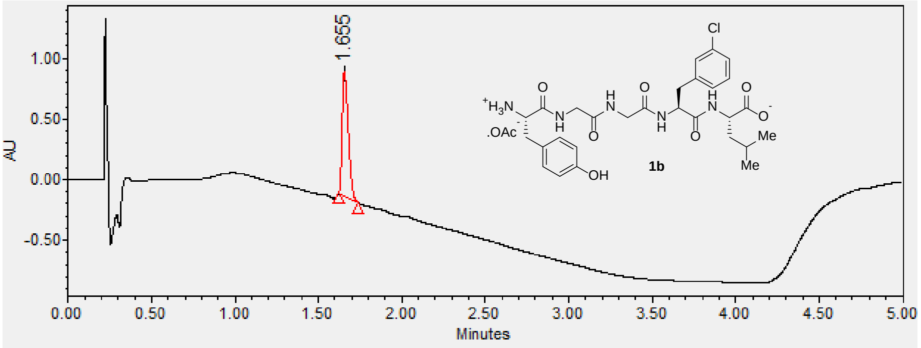

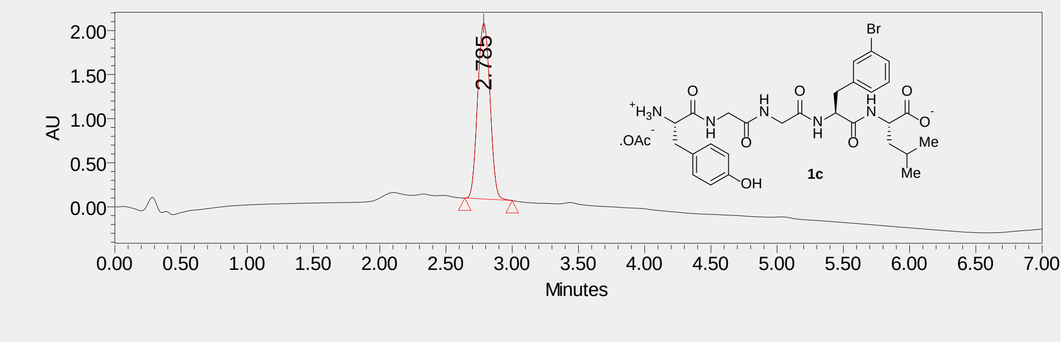

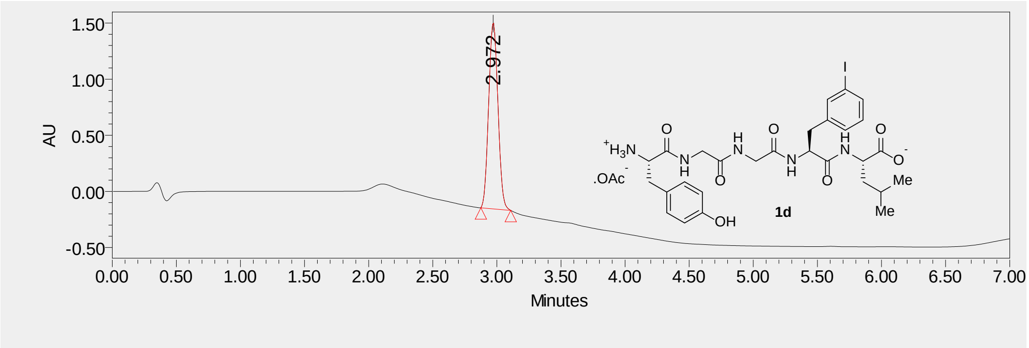
AU
1.50
1.00
0.50
0.00
-0.50
2.972
0.00 0.50 1.00 1.50 2.00 2.50 3.00 3.50 4.00 4.50 5.00 5.50 6.00 6.50 7.00
Minutes
I
+H3N
.OAc-
OH
Me
Me
1d

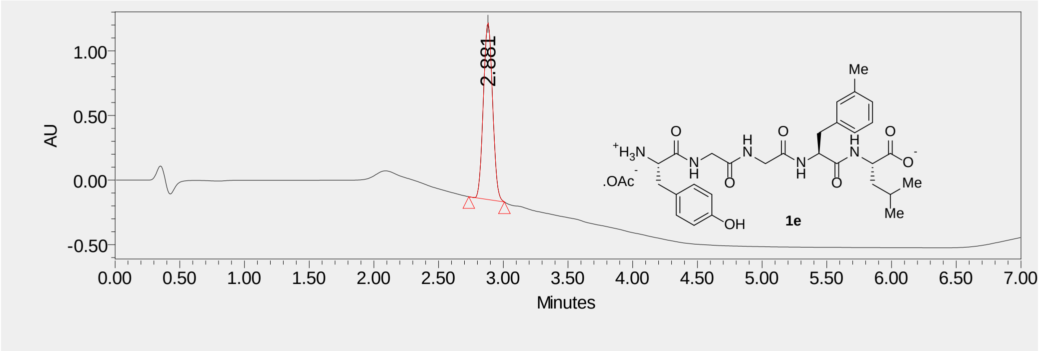
AU
1.00
0.50
0.00
-0.50
2.881
0.00 0.50 1.00 1.50 2.00 2.50 3.00 3.50 4.00 4.50 5.00 5.50 6.00 6.50 7.00
Minutes
Me
+H3N
.OAc-
OH
Me
Me
1e

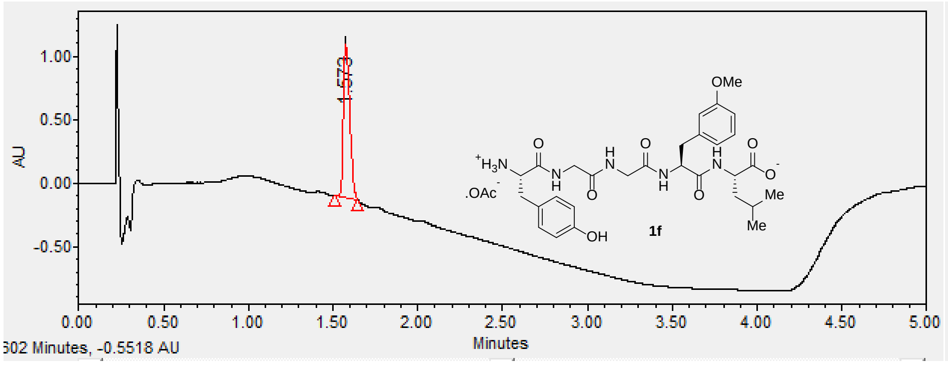
AU
1.00
0.50
0.00
-0.50
1.573
0.00 0.50 1.00 1.50 2.00 2.50 3.00 3.50 4.00 4.50 5.00
Minutes
OMe
+H3N
.OAc-
OH
Me
Me
1f

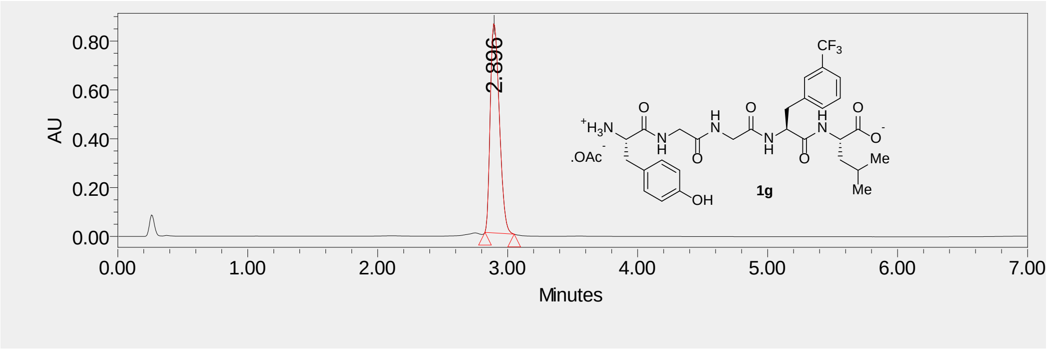
0.80
0.60
0.40
0.20
0.00
AU
2.896
0.00
1.00
2.00
3.00
4.00
5.00
6.00
7.00
Minutes
1g

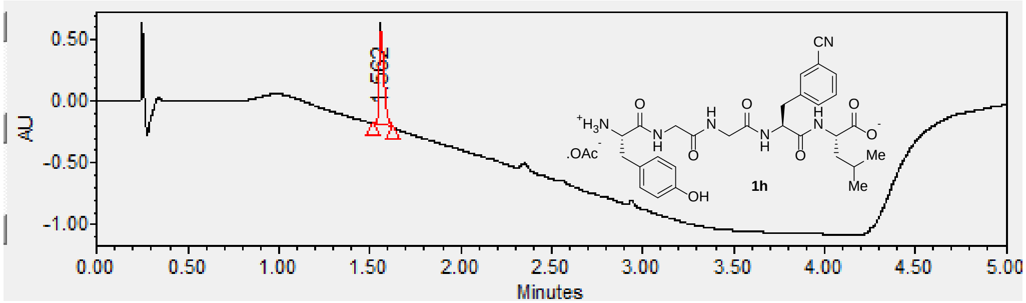
0.50
0.00
-0.50
-1.00
AU
0.00
0.50
1.00
1.50
2.00
2.50
3.00
3.50
4.00
4.50
5.00
Minutes
1h

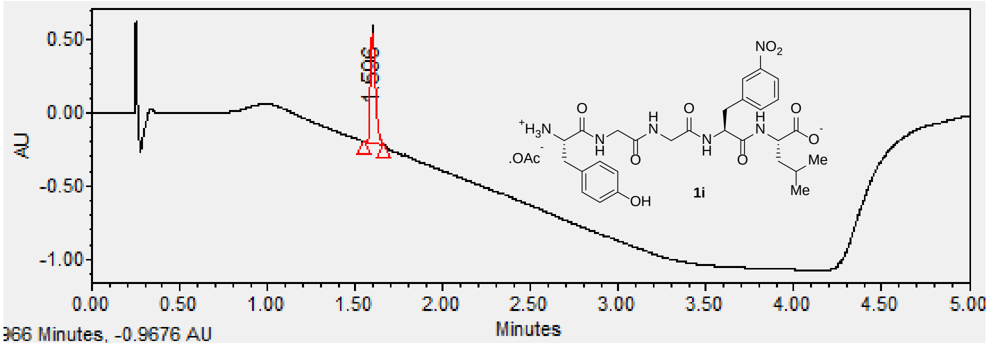
0.50
0.00
-0.50
-1.00
AU
0.00
0.50
1.00
1.50
2.00
2.50
3.00
3.50
4.00
4.50
5.00
Minutes
966 Minutes, -0.9676 AU
1i

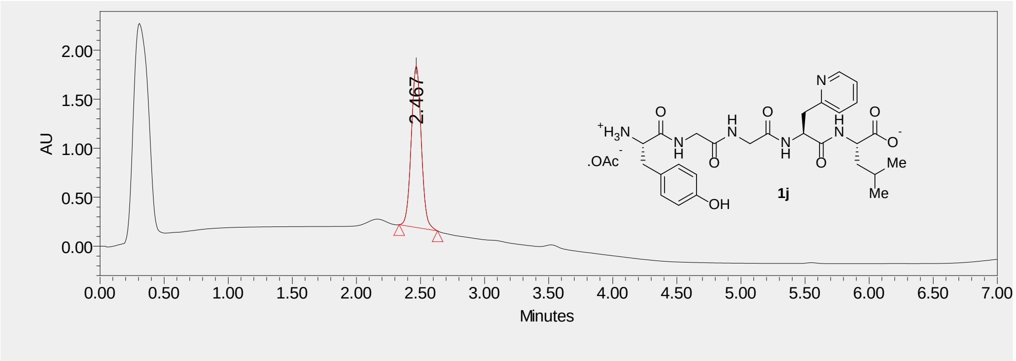
2.467
AU
Minutes
1j
.OAc
OH
Me

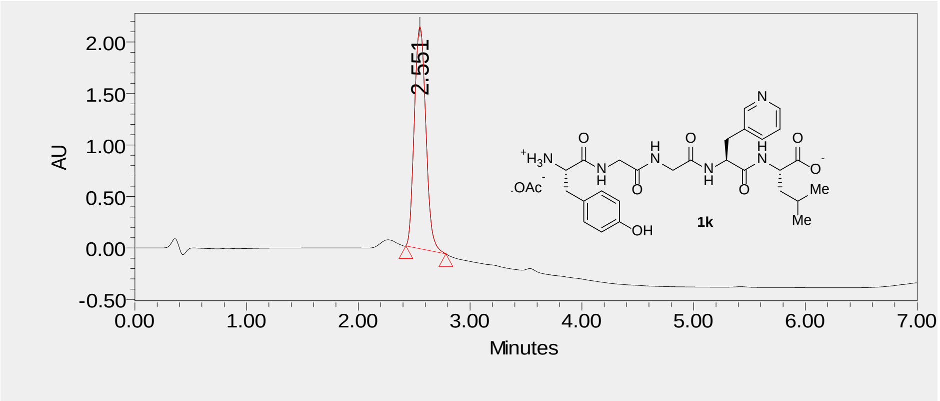
2.551
AU
Minutes
1k
.OAc
OH
Me

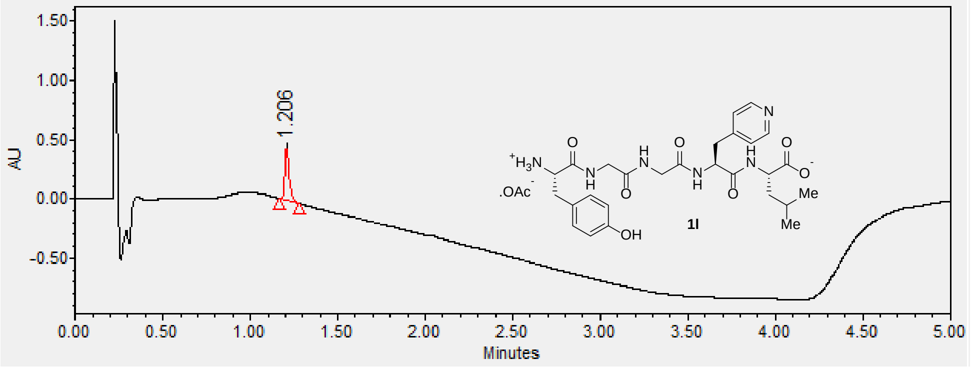
1.206
AU
Minutes
1l
.OAc
OH
Me
